# Distinct receptors for a promiscuous RFamide neuropeptide co-regulate a distributed circuit for locomotory arousal in *C. elegans*

**DOI:** 10.1093/genetics/iyag188

**Published:** 2026-07-18

**Authors:** Nathan De Fruyt, Aleksandra Chudinova, Saurabh Thapliyal, Ellen Geens, Charline Roy, Elke Vandewyer, Sara Van Damme, Lucas Mergan, Isabel Beets

**Affiliations:** Department of Biology, University of Leuven (KU Leuven), Leuven 3000, Belgium; Department of Biology, University of Leuven (KU Leuven), Leuven 3000, Belgium; Department of Biology, University of Leuven (KU Leuven), Leuven 3000, Belgium; Neurobiology Division, MRC Laboratory of Molecular Biology, Cambridge CB2 0QH, United Kingdom; Department of Biology, University of Leuven (KU Leuven), Leuven 3000, Belgium; Department of Biology, University of Leuven (KU Leuven), Leuven 3000, Belgium; Department of Biology, University of Leuven (KU Leuven), Leuven 3000, Belgium; Department of Biology, University of Leuven (KU Leuven), Leuven 3000, Belgium; Department of Biology, University of Leuven (KU Leuven), Leuven 3000, Belgium; Department of Biology, University of Leuven (KU Leuven), Leuven 3000, Belgium

**Keywords:** neuropeptide, ligand promiscuity, *C. elegans*, locomotory arousal, Animalia

## Abstract

Neuropeptides modulate brain function by acting as ligands for a wide range of receptors. Systematic pharmacological screening efforts revealed extensive promiscuity in peptide-receptor pairs. However, the endogenous receptors and functions of promiscuous neuropeptides remain poorly understood. Here, we investigated how the *Caenorhabditis elegans* promiscuous RFamide-peptide FLP-14 and its receptors modulate a circuit for O_2_-evoked locomotory arousal. We show that FLP-14 peptides released from O_2_-sensing neurons and interneurons attenuate O_2_-evoked arousal by acting on 3 phylogenetically diverse G protein-coupled receptors (DMSR-3, FRPR-8, and NPR-40). Loss of each receptor individually confers strong hyperarousal by O_2_ comparable to the loss of *flp-14.* Distinct FLP-14 receptors have nonredundant roles in different target cells, including RID and RIM interneurons, that correlate antagonistically with locomotory arousal. Our results show that FLP-14 signaling requires coordinated modulation of multiple circuit nodes through distinct receptors in attenuating O_2_-evoked arousal. This promiscuous RFamide peptide network is reminiscent of monoaminergic neuromodulatory networks, which shape behavior through multiple receptors that differentially regulate distributed target neurons.

## Introduction

Neuropeptidergic signaling is widespread and plays essential roles in all nervous systems. Neuropeptides predominantly signal by binding to G protein-coupled receptors (GPCRs), and many studies reveal complexity in their receptor-ligand interactions. Indeed, systematic pharmacological screens across animals showed that individual neuropeptides can activate 1 to many GPCRs, which can include evolutionarily related as well as phylogenetically diverse receptors (Williams et al. 2017; Pándy-Szekeres et al. 2018; Foster et al. 2019; Jékely 2021; Beets et al. 2023; Hevesi et al. 2024; Thiel et al. 2024). The human prolactin-releasing peptide (PrRP), for example, can bind to 6 receptors, including ligand-specific and promiscuous receptors of diverse receptor families (Jékely 2013; Mirabeau and Joly 2013; Foster et al. 2019), and endogenous opioid peptides bind to both classical opioid receptors and unrelated chemokine receptors (Ikeda et al. 2013; Meyrath et al. 2020; Wang et al. 2023). The endogenous roles of promiscuous neuropeptide signaling are, however, not well understood in vivo.

RFamide-type peptides are among the most ancient groups of neuropeptides and have evolved complex patterns of receptor interactions (Walker et al. 2009; Pedragosa-badia et al. 2013; Elphick and Mirabeau 2014; Leprince et al. 2017; Pándy-Szekeres et al. 2018; Foster et al. 2019; Bhat et al. 2021; Beets et al. 2023; Kovács et al. 2024; Thiel et al. 2024). Where studied, RFamide peptides commonly activate multiple rhodopsin-type GPCRs and have been implicated in many biological processes (Walker et al. 2009; Li and Kim 2014; Peymen et al. 2014; Zatylny-Gaudin and Favrel 2014; Ma et al. 2017; Ubuka and Tsutsui 2018; Kovács et al. 2024). GPCRs that are pharmacologically activated by the same RFamide peptide can be found in different branches of the rhodopsin phylogenetic tree, similar to receptors for monoamines (Sheau et al. 2002; Liu et al. 2003; Carre-Pierrat et al. 2006; Cardoso et al. 2012; Yamamoto et al. 2012; Tierney 2018; Beets et al. 2023). This suggests active evolutionary selection of receptor-ligand promiscuity and makes the RFamide system an attractive model to investigate how promiscuous neuropeptide signaling modulates neural circuits and behavior.

In *Caenorhabditis elegans*, the neuropeptide-receptor network has been comprehensively mapped across all neuron classes, providing a suitable model for systems studies (Taylor et al. 2021; Beets et al. 2023; Ripoll-Sánchez et al. 2023). Through large-scale receptor deorphanization, we previously identified the RFamide FLP-14 as a promiscuous neuropeptide that, like RFamides in mammals, exhibits crosstalk with multiple receptor types (Beets et al. 2023). Pharmacological screens identified 12 FLP-14 receptors, comprising both evolutionarily related and phylogenetically diverse GPCRs, that are expressed widely across the nervous system (Olson and the CeNGEN Consortium; Gershkovich et al. 2019; Beets et al. 2023; Ripoll-Sánchez et al. 2023; Marquina-Solis et al. 2024; Pradhan et al. 2025; P.Z. Wang et al. 2025). FLP-14 signaling plays important roles in *C. elegans* behavior ranging from the modulation of forward motor state to sensory-guided behaviors (Lim et al. 2016; Marques et al. 2021; P.Z. Wang et al. 2025). Previous studies identified 2 FLP-14 receptors that act in distinct circuits for mechanosensory nociception (FRPR-19) and salt aversive learning (FRPR-10). Whether FLP-14 signaling requires additional receptors to regulate behavior has not yet been determined.

While distinct receptors mediate pleiotropic functions of FLP-14 in behavior (Marques et al. 2021; P.Z. Wang et al. 2025), single-cell RNA sequencing studies revealed expression of multiple FLP-14 receptors in local microcircuits, which suggests additional roles for ligand promiscuity. The core circuit driving aerotaxis behavior, in particular, shows enriched expression of transcripts for *flp-14* and its receptors (Sun and Hobert 2021; Taylor et al. 2021; Ripoll-Sánchez et al. 2023), although a role of FLP-14 signaling in O_2_-guided behavior is not known.

*C. elegans* dwell locally at low (7%) O_2_ concentrations and avoid higher (12% to 21%) O_2_ levels (Gray et al. 2004). An increase from 7% to 21% O_2_ also induces a state of persistent locomotory arousal (Coates and de Bono 2002; Cheung et al. 2005; Chang et al. 2006; Rogers et al. 2006; Zimmer et al. 2009; Busch et al. 2012; Laurent et al. 2015; Li et al. 2020). This increase in O_2_ concentration is detected by sensory neurons called URX, AQR, and PQR, while BAG sensory neurons are active at low (7%) O_2_ levels (Cheung et al. 2005; Rogers et al. 2006; Busch et al. 2012; Nichols et al. 2017). Arousal by 21% O_2_ is mainly driven by URX and interneurons that are postsynaptic, called RMG, and is modulated by neuropeptide signaling from these neurons (Macosko et al. 2009; Busch et al. 2012; Laurent et al. 2015; Li et al. 2020). Expression analyses using single-cell transcriptomics and fluorescent reporters highlight expression of *flp-14* and its receptors in URX, AQR, PQR, and BAG, as well as in RMG and other downstream interneurons (Fig. 1, a and b), suggesting that FLP-14 may regulate O_2_-evoked arousal through concerted actions in multiple circuit nodes (Olson and the CeNGEN Consortium; Sun and Hobert 2021; Ripoll-Sánchez et al. 2023; Marquina-Solis et al. 2024; Pradhan et al. 2025).

**Fig. 1.**
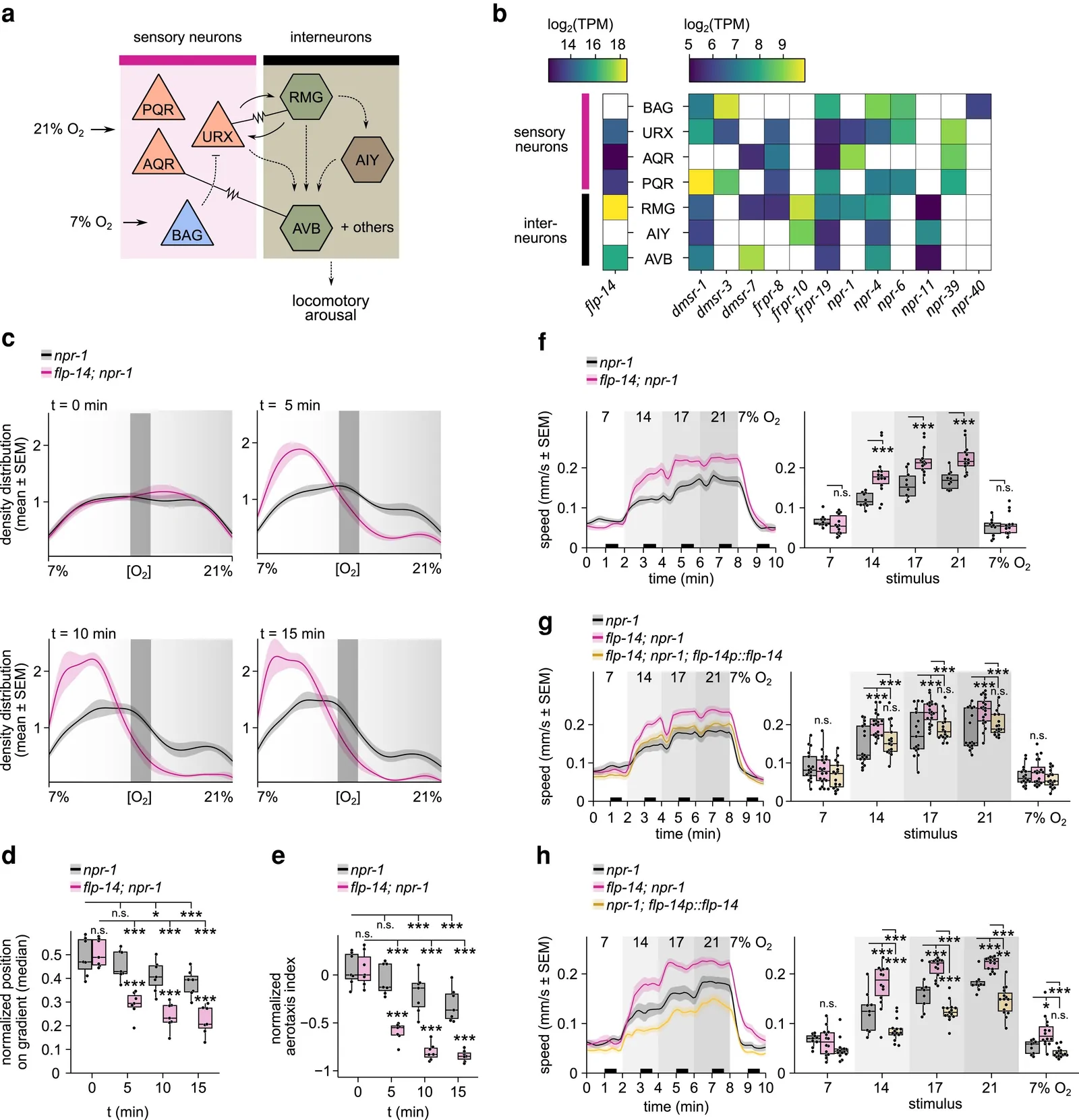
FLP-14 attenuates avoidance and locomotory arousal in response to aversive O_2_. a) Schematic of the core circuit regulating behavioral responses to environmental changes in O_2_ levels. URX, AQR, and PQR are activated by high (21%) O_2_, while BAG is activated by low (7%) O_2_. RMG, AIY and AVB interneurons integrate O_2_-sensing with input from other sensory neurons to orchestrate locomotory arousal (Zimmer et al. 2009; Busch et al. 2012; Laurent et al. 2015; Hussey et al. 2018; Cook et al. 2019). b) Genes encoding FLP-14 peptides and 12 known FLP-14 receptors are expressed in neurons of the core O_2_ circuit (Olson and the CeNGEN Consortium). c) Distribution of *flp-14* mutants and *npr-1* controls that were grown at 7% O_2_ on a continuous 7% to 21% O_2_ gradient after 0, 5, 10, and 15 min. The distribution of animals across the gradient is represented as average density across replicates ± SEM. d) *flp-14* mutants on average locate to a lower position on the 7% to 21% O_2_ gradient throughout the assay. Median position of animals on the gradient is normalized to the behavioral arena (0 = 7% O_2_ border, 1 = 21% O_2_ border). e) *flp-14* mutants show more negative aerotaxis indexes than *npr-1* controls after 5, 10, and 15 min. Aerotaxis index = ((number of animals at high O_2_) – (number of animals at low O_2_))/(total number of animals), excluding animals at the origin (shaded region). f) The absolute speed at aversive O_2_ levels is increased in *flp-14* mutants as compared to *npr-1* control animals when acclimated to 7% O_2_. g) Reintroducing a wild-type *flp-14* transgene under control of the endogenous promoter sequence in *flp-14* mutants restores the absolute speed at aversive O_2_ levels. h) Overexpression of *flp-14* reduces speed at aversive O_2_ concentrations. For aerotaxis assays, *n* = 7 on 4 d. Median positions on the gradient were compared between strains and time points by 2-way ANOVA on a GLMM considering paired measures per replicate. Aerotaxis indexes are normalized to the aerotaxis index at *t* = 0 min and compared by Kruskal–Wallis and post hoc pairwise Wilcoxon rank sum tests. All relevant comparisons were corrected using Holm–Bonferroni correction. Average speed over time is depicted as smoothed mean values (mm/s) ± SEM; *n* ≥ 9 assays on at least 3 d; intervals for which values were averaged in statistical comparison are indicated as black lines on the trace panels; ANOVA was performed on a GLMM considering paired measures per replicate and were corrected for all relevant comparisons using Holm–Bonferroni correction; *P*-values ns = not significant; * < 0.05; ** < 0.01; *** < 0.005.

Here, we show that FLP-14 signaling opposes locomotory arousal by O_2_ through a promiscuous network of phylogenetically diverse receptors. FLP-14 modulates multiple cells in the arousal circuit by signaling to 3 diverse GPCRs—DMSR-3, FRPR-8, and NPR-40. Disrupting any component of this signaling network confers strong hyperactivity in response to aversive O_2_. Our results reveal a promiscuous RFamide system that counteracts hyperarousal by signaling to distributed, including antagonistic, neurons through different receptors.

## Materials and methods

### Animals

Animals were maintained in standard conditions at 20 °C on nematode growth medium (NGM) plates seeded with *Escherichia coli* strain OP50 (Stiernagle 2006). When acclimated at 7% O_2_, animals were grown for 1 generation in a hypoxic chamber (Coy laboratory products), controlled for atmosphere, temperature, and relative humidity. Animals acclimated to 21% O_2_ were cultivated in temperature-controlled conditions. All animals used in this study were synchronized to be in the young adult stage by transferring fourth larval stage (L4) animals to a fresh culture plate 18 h before experiments (Altun and Hall 2009). The *npr-1(ad609)* reference allele was crossed into our lab's wild-type N2 strain 6 times. The *flp-14(gk1055)* allele was used as loss-of-function in all figures, unless stated otherwise (Supplementary Fig. 1, i and j). A detailed list of all strains used in this study can be found in the Reagent Table (Supplementary Table 1).

### Molecular biology and transgenesis

Plasmids used in this study are listed in the Reagent Table (Supplementary Table 1). Open reading frames and promoters were derived from predictions curated on WormBase (http://wormbase.org, version WS240; Davis et al. 2022; Sternberg et al. 2024). Most plasmids were cloned using Multisite Gateway Three-Fragment cloning system (Invitrogen) into the pDESTR4R3 II backbone. We used the promoters *gcy-32p* (0.6 kb) (Gray et al. 2004; Cheung et al. 2005), *flp-8p* (2.7 kb) (Cheung et al. 2005), *nlp-56p* (2.9 kb) (Lorenzo et al. 2020; Taylor et al. 2021), *flp-5p* (2.3 kb) (Kim and Li 2004; Chen et al. 2017), *ceh-10p* (3.6 kb) (Svendsen and McGhee 1995; Lim et al. 2016), *C39H7.2p* (1.4 kb) (Taylor et al. 2021), *myo-2p* (0.9 kb) (Okkema et al. 1993), *flp-17p* (3.3 kb) (Gray et al. 2004; Cheung et al. 2005), and *tdc-1p* (3.4 kb) (Alkema et al. 2005). Cell types targeted by these promoters are listed in Supplementary Table 2.

*frpr-8p* (1.9 kb), *dmsr-3p* (3 kb), and the *frpr-8* and *dmsr-3* gDNA and cDNA sequences were PCR amplified from mixed stage N2 genomic DNA and cloned into the pDONR P4-P1 or pDONR 221 backbone. *npr-40p* (3 kb) was cloned into a PCR amplified *sl2::gfp::let-858 3′UTR* expression backbone, after which the *npr-40* gDNA sequence was cloned in between the promoter and *sl2* spliced leader sequences, using NEBuilder HiFi DNA Assembly (New England Biolabs) of PCR amplified sequences. *dmsr-3* cDNA was cloned in a PCR amplified expression backbone by NEBuilder DNA assembly, in between the *flp-17p* promoter sequence and the *sl2::gfp::tbb-2 3′UTR* reporter cassette.

Recombinant plasmids were micro-injected in the syncytial gonads of young adults together with a co-injection marker and carrier DNA. All plasmids included a co-expression marker downstream of the gene of interest (Supplementary Table 1) and were injected at concentrations that yielded bright co-expression marker fluorescence intensity. Concentrations of plasmid and co-injection marker are indicated in the Reagent Table (Supplementary Table 1). Carrier DNA (1Kb Plus DNA Ladder, Invitrogen) was injected at 50 ng/µl.

The *flp-14(ibt25)* allele was created by CRISPR-Cas9 gene editing using crRNA sequences (cguuucggacgcaaauaaga and gcguggcuugguagagcucg) targeting PAM sites close to the start and stop codons of the *flp-14* gene sequence, and a homologous repair template consisting of a concatenated sequence of the regions (2 × 75 bp) flanking the intended PAM sites, in a co-CRISPR approach using *dpy-10* as a marker for screening (Dokshin et al. 2018). The deletion allele was crossed out 3 times after CRISPR editing. *flp-14(syb8434), dmsr-3(syb9311), frpr-8(syb9277),* and *npr-40(syb10826)* transcriptional reporter strains were generated using CRISPR-Cas9 to insert the *sl2::gfp::h2b* reporter cassette before the STOP codon of the respective gene.

An extrachromosomal array driving expression of the YC2.60 Ca^2+^ indicator in RMG in the strain AX3436 (*npr-1(ad609); dbIs637[ncs-1p::Cre; flp-21p::LoxP-STOP-LoxP-YC2.60; unc-122p::RFP*] (Fenk and de Bono 2015)) was integrated in the genome by UV irradiation, as described before (Evans 2006). Briefly, 100 L4 stage animals were transferred to unseeded NGM plates and exposed to 0.030 J/cm^2^ UV irradiation using a UV transilluminator (Stratalinker 1800). A stably integrated hit was outcrossed 4 times.

### Behavioral assays

*Aerotaxis assays.* Aerotaxis was assayed by examining the population distribution across a continuous 7% to 21% O_2_ gradient (Gray et al. 2004; Cheung et al. 2005; Rogers et al. 2006; Bretscher et al. 2008; Li et al. 2020). A total of 60 to 100 animals were placed in the center of a rectangular patch of *E. coli*  OP50 grown in 2xYT medium (40 µl), seeded on an NGM plate (Ø 55 mm) 16 h before the assay. Animals were left at the microscope to dishabituate from mechanical stimulation for 10 min after transferring to the assay plate. Subsequently, the local environment was sealed with a 33 × 15 mm polydimethylsiloxane (PDMS) chip with 1 inlet for the 7% O_2_ mixture, 1 inlet for the 21% O_2_ mixture, and 2 matching outlets on either side of the chip. Humidified gas flow was controlled at 3.0 ml/min by a PHD 2000 Infusion syringe pump (Harvard apparatus). O_2_ gas mixtures for all O_2_ concentrations were balanced with N_2_ (AirLiquide). Videos were recorded in the DinoCapture 2.0 software at 2 frames per second from a Dino-Lite AM4023CT camera mounted on either a Leica M60 carrier with a 0.32x video objective (Leica) or Nikon SMZ745T optics carrier with a 0.5 achromat objective (Nikon), both fitted onto a Nikon C-DS diascopic stand with led illumination. The position of animals and the behavioral arena was determined in Fiji (ImageJ), and compared at 0, 5, 10, and 15 min or 0 and 15 min after the recording started.

Replicates were blinded before analysis. The position of each animal on the gradient was normalized against the edges of the behavioral arena. Each strain was tested on 3 to 4 different days, having 1 to 2 individual replicates per strain per day. Aerotaxis indexes were calculated as described (Li et al. 2020) by dividing the area in 9 equal sections along the gradient and computing ((the number of animals in the high O_2_ area) − (the number of animals in the low O_2_ area))/(total number of animals), excluding animals at the central section of the gradient (shaded area in Fig. 1c, Supplementary Fig. 1a). Median positions on the gradient were compared between strains and time points by 2-way ANOVA on a generalized linear mixed model (GLMM) considering paired measures per replicate. Aerotaxis indexes were normalized by subtracting the median aerotaxis index at *t* = 0 for each strain. Since parametric model assumptions were violated, we evaluated statistical significance by Kruskal–Wallis and post hoc pairwise Wilcoxon rank sum tests, adjusting for multiple comparisons on relevant comparisons by the Holm–Bonferroni method.

*Locomotory responses to O_2_*. Behavioral responses to aversive O_2_ were assayed as described (Fenk and de Bono 2015; Laurent et al. 2015; Beets et al. 2020). Animals grown at 7% O_2_ were used in all behavioral assays, unless stated otherwise. Measurements were averaged across animals per assay plate (biological replicate), for at least 6 replicates across 2 or more assay days. A total of 20 to 30 animals were picked onto an NGM plate (Ø 55 mm) seeded with a square (2 cm × 2 cm) patch of *E. coli*  OP50 grown in 2xYT medium (20 µl) 16 h before the assay. The local atmosphere was sealed by a 12 × 12 mm arena in a PDMS chip, connected by 4 inlets to gas-filled syringes. Humidified gas flow was controlled at 3.0 ml/min by a PHD 2000 Infusion syringe pump (Harvard apparatus) and regulated by an 8-pinch-valve Valvebank II unit (AutoMate Scientific) for automated switching between gas mixtures. Worms were left to dishabituate from mechanical stimulation during picking for 8 to 10 min at atmospheric O_2_. Animals were exposed to 7% O_2_ for 2 min before recording started. Recording lasted for 10 min, switching gas mixtures (AirLiquide) every 2 min. Videos were recorded on the same setup as for aerotaxis assays and were processed using ZenTracker software (https://github.com/wormtracker/zentracker) (Laurent et al. 2015), extracting behavioral parameters including locomotion speed, proportion of animals reversing, and proportion of animals performing omega turns from animal traces through time. Measurements of animals inside the food patch were included, but not when animals collided. For quantification and analyses, refer to statistical analysis section and figure legends.

Videos were processed by an automated algorithm (ZenTracker) and R script. Each strain was tested on at least 2 different days, having 2 to 5 individual replicates per strain per day. Speed measures were averaged per biological replicate (assay plate) over a 1-min interval from 40 s after switching stimuli till 20 s before switching to the next stimulus interval to exclude acceleration and deceleration when switching stimuli. To capture turning and reversals, these measures were averaged over a 1-min interval from 20 s till 80 s after switching stimuli. A full-factorial GLMM was used to predict statistical significance, in which strain and O_2_ stimulus were considered fixed factors. Measurements on the same replicates across stimulus intervals were paired by considering replicate ID as a random factor. The impact of day-to-day differences was evaluated by visualizing residuals in a Q–Q plot and based on differences in Akaike Information Criterion. When apt, the assay day was considered as an additional random factor. ANOVA was followed by pairwise post hoc testing for the relevant comparisons. Holm–Bonferroni correction was applied to *P*-values to correct for multiple comparisons, including comparisons to negative and positive controls and testing multiple strains across figure panels. Thus, *P*-values were corrected for screening multiple receptor mutants to controls in Fig. 3, a to d and Supplementary Fig. 3, a to c, as well as comparing multiple cell-specific rescue strains across panels for *flp-14* and each receptor mutant to positive and negative controls in Figs. 2, b to g, and 5, and Supplementary Fig. 3, d and e.

**Fig. 2.**
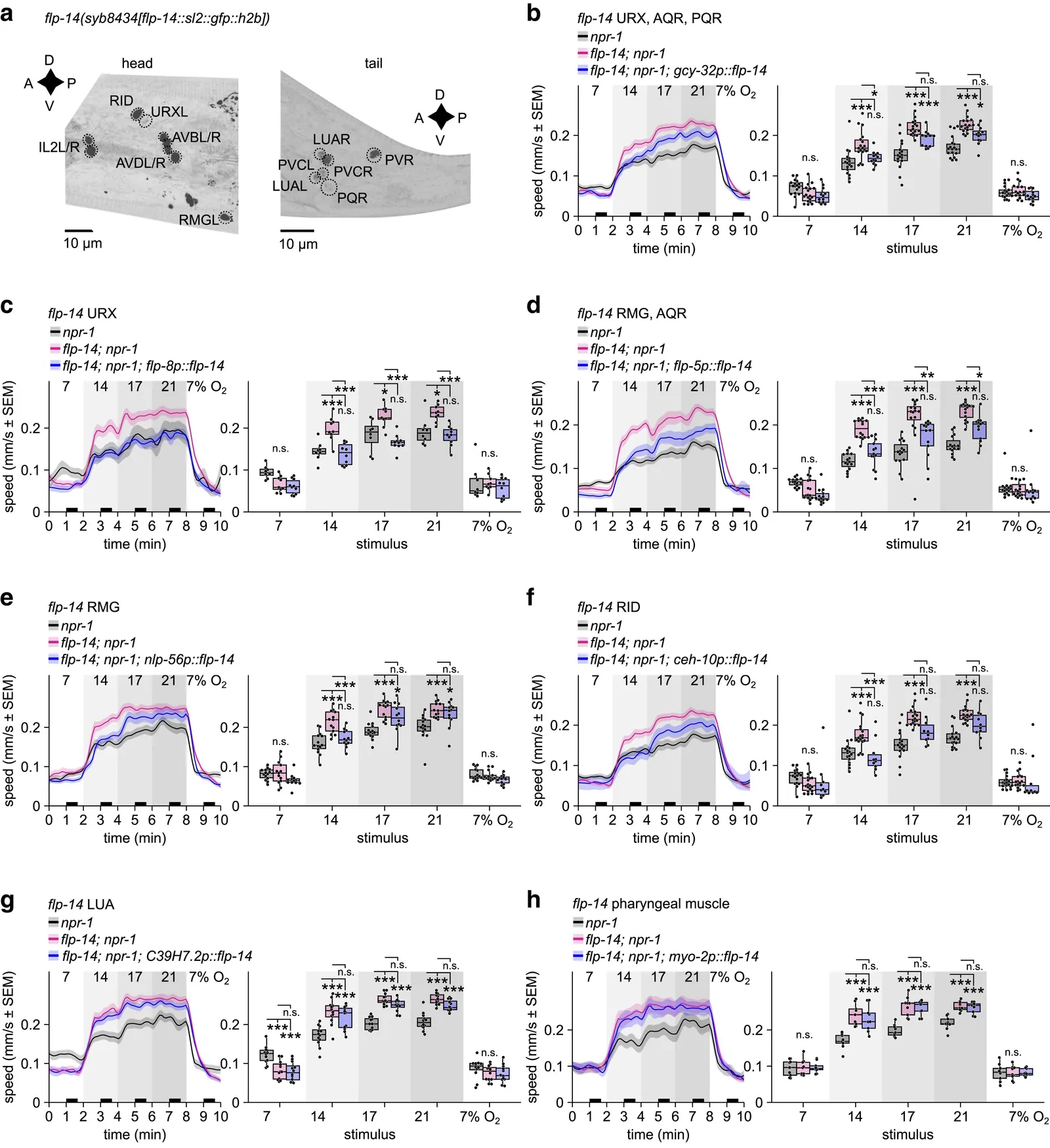
Release of FLP-14 from O_2_-sensory neurons and interneurons attenuates O_2_-evoked arousal. a) Expression pattern of a transcriptional *flp-14* reporter by CRISPR insertion of a fluorescent reporter with nuclear localization (*sl2::gfp::h2b*) in the endogenous *flp-14* locus of *npr-1* animals grown at 7% O_2_. D, dorsal; V, ventral; A, anterior; P, posterior. b) Restoring *flp-14* expression in the O_2_-sensing neurons URX, AQR, and PQR partially rescues the enhanced speed at aversive O_2_ levels in *flp-14* mutants. c) Restoring *flp-14* expression in URX restores absolute speed at aversive O_2_ levels in *flp-14* mutants. d) Expressing wild-type *flp-14* under control of a *flp-5* promoter, driving expression in RMG and AQR, among other neurons (Supplementary Table 2), restores arousal by O_2_ in *flp-14* mutants. e and f) Cell-specific expression of *flp-14* in RMG or RID interneurons partially rescues the arousal phenotype in *flp-14* mutants. g and h) Cell-specific expression of *flp-14* in LUA neurons, or ectopic expression in pharyngeal muscles could not attenuate hyperarousal in *flp-14* mutants; see Supplementary Table 2 for an overview of promoter sequences and expression patterns. Average speed over time is depicted as smoothed mean values per strain (mm/s) ± SEM; for (b to h), *n* ≥ 8 on at least 2 d; intervals for which values were averaged in statistical comparison are indicated as black lines in the speed over time panels; ANOVA was performed on a GLMM considering paired measures per replicate and were corrected for all relevant comparisons using Holm–Bonferroni correction. *P*-values ns = not significant; * < 0.05; ** < 0.01; *** < 0.005.

**Fig. 3.**
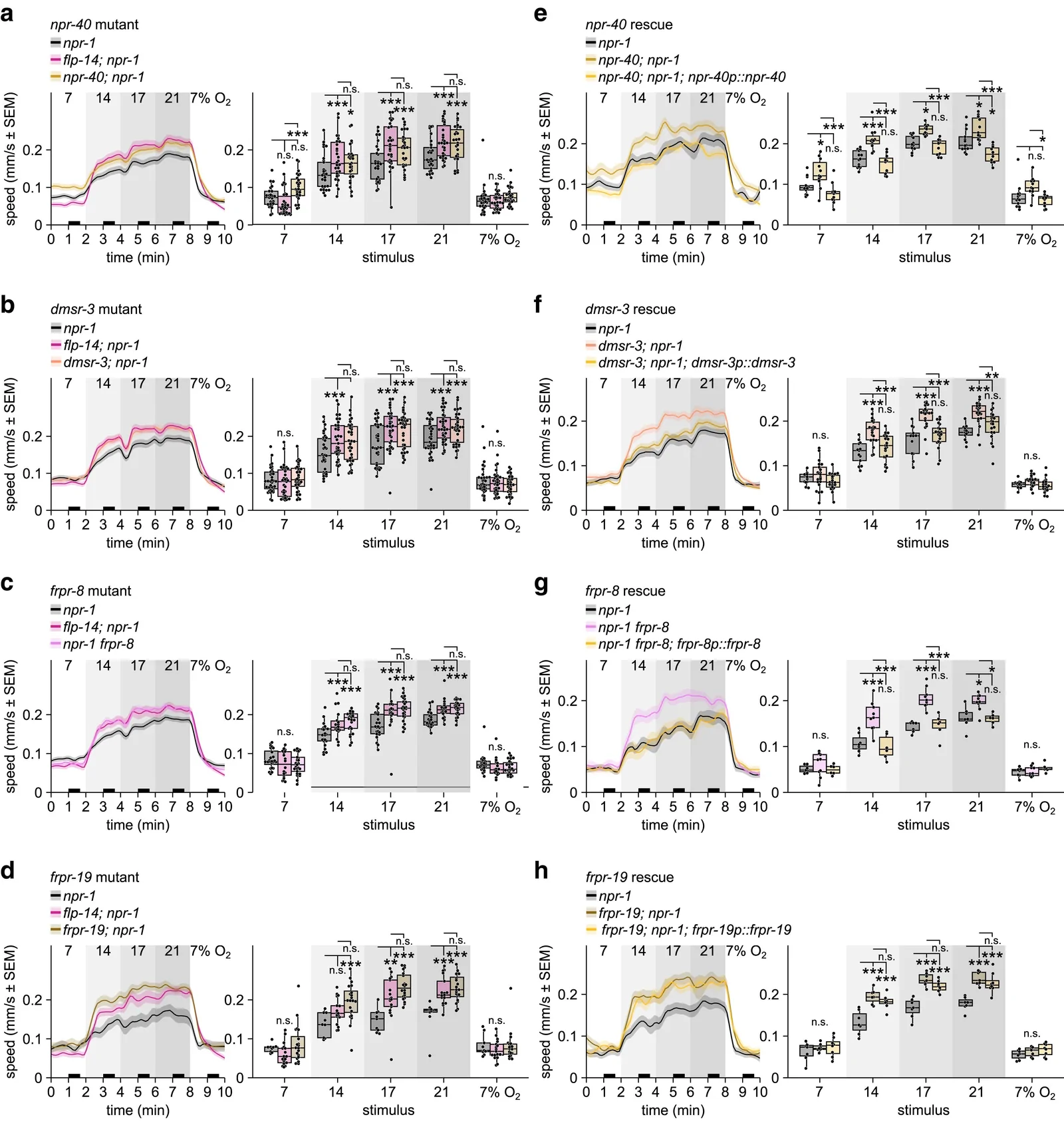
Three FLP-14 G protein-coupled receptors, FRPR-8, DMSR-3, and NPR-40, attenuate locomotory arousal by O_2_. a to d) Mutants for *npr-40*, *dmsr-3*, *frpr-8*, and *frpr-19* phenocopy *flp-14* animals in O_2_ response assays. e to g) Reintroducing wild-type copies of *npr-40*, *dmsr-3*, and *frpr-8* restores the absolute speed at aversive O_2_ levels in respective receptor mutants. h) Expressing a wild-type *frpr-19* transgene, under control of the endogenous promoter (Marques et al. 2021), fails to rescue the mutant phenotype. Average speed over time is depicted as smoothed mean values (mm/s) ± SEM; *n* ≥ 8 on at least 3 d; intervals for which values were averaged in statistical comparison are indicated as black lines on the speed traces; ANOVA was performed on a GLMM considering paired measures per replicate and were corrected for all relevant comparisons and testing of multiple receptors across figure panels using Holm–Bonferroni correction; *P*-values ns = not significant; * < 0.05; ** < 0.01; *** < 0.005.

### Calcium imaging

Ca^2+^ levels were imaged in immobilized animals as described before (Beets et al. 2020). Imaging was done within 30 min after the animals were transferred from the cultivation incubator kept at 7% O_2_. Animals were glued to a 2% agarose (low gelling temperature; Sigma) pad supplemented with 1 mM CaCl_2_ using Dermabond tissue adhesive (Ethicon), leaving the nose unblocked, and supplemented with a drop of *E. coli*  OP50 suspended in M9 buffer at the nose to prevent food deprivation. Animals were sealed from the environment during recording using a 200-µM deep PDMS chip connected to syringes containing the tested gas mixtures. Gas flow was kept constant at 3.0 ml/min by a 2000 PHD Infusion Syringe pump (Harvard Apparatus) and gated by a 4-pinch-valve unit (Automate Scientific) for automated switching between gases. Before recording, animals were allowed to re-acclimate to the 7% O_2_ environment for 2 min, and to pulsed excitation-wavelength illumination for 1 min. Image streams were acquired on a Zeiss AxioObserver Z1 microscope equipped with a 40× objective and connected to an ORCA-Flash4.0 V2 camera (Hamamatsu) via W-VIEW GEMINI image splitting optics (Hamamatsu) for simultaneous recording of both YFP and CFP emission wavelengths. Images were acquired at 2 frames per second in MetaMorph software (Molecular Devices). Videos were processed using Neuron Analyzer in single neuron mode (Laurent et al. 2015).

Each strain was tested on at least 7 different days. Measures were averaged per biological replicate (assay plate) over a 1-min interval from 40 s after stimulus onset till 20 s before stimulus offset. A full-factorial GLMM was applied in which strain and O_2_ stimulus were considered fixed factors. Measures on the same replicates across stimulus intervals were paired by considering replicate number as a random factor. We applied a log(1 + *x*) transformation to approximate normal distribution of model residuals. ANOVA was followed by post hoc testing on relevant comparisons for significant fixed-factor effects. Holm–Bonferroni correction was applied to *P*-values to account for the appropriate number of relevant comparisons.

### Confocal microscopy and image analysis

Confocal images were acquired on an LSM900 Zeiss inverted confocal microscope setup. Young adult hermaphrodites acclimated to 7% O_2_ were immobilized in NaN_3_ (0.5 M) on a thin 2% agarose pad. Imaging was performed within 30 min after removing animals from their 7% O_2_ culture environment to avoid re-acclimation to atmospheric O_2_. Identification of receptor-expressing neurons in the NeuroPAL landmark strain was confirmed in at least 20 animals. The identity of neurons in the *flp-14* reporter strain was based on previously established anatomical position (Sun and Hobert 2021; Ripoll-Sánchez et al. 2023). The position of URX and PQR cell bodies was further confirmed by co-localized expression of a fluorescent marker (*gcy-32p::tagBFP*).

For quantification of nuclearly localized reporter expression for *flp-14, flp-8,* and *flp-17*, maximum intensity projections along the *z* axis were generated and quantified in Fiji (ImageJ). To account for background autofluorescence, the average intensity of 3 randomly selected nearby nonnuclear regions of the same dimensions was calculated and subtracted from intensity values for each identified neuronal nucleus. We compared background-corrected intensity values in Supplementary Fig. 5, d and e to controls by Student’s *t*-test. Comparisons between 2 < strains in Supplementary Fig. 5, g and h were done using ANOVA on log(1 + *x*) transformed values, to meet normality criteria. Because parametric model assumptions were violated in Supplementary Fig. 6, g and h, we compared background-corrected intensity values of mutants to controls using Kruskal–Wallis statistics and post hoc pairwise Wilcoxon rank sum testing. Holm–Bonferroni correction was applied to adjust for all relevant comparisons.

### Statistical tests

All statistical analyses were performed using R 4.5.0 in RStudio (posit.co), unless stated otherwise. Normality criteria were examined numerically by comparing multiple test metrics (Shapiro–Wilk's, Anderson–Darling, Kolmogorov–Smirnov, and D’Agostino & Pearson) and evaluated visually by histogram, normal Q–Q plot, and by plotting residuals and fitted values for each observation. Homogeneity of variance was tested using Levene's test and corrected for when significantly different between samples.

## Results

### Loss of the RFamide neuropeptide FLP-14 enhances locomotory arousal by O_2_

Transcripts encoding FLP-14 and 12 reported FLP-14 receptors are expressed in the main O_2_-sensing neurons and in interneurons controlling aerotaxis behavior (Fig. 1, a and b) (Olson and the CeNGEN Consortium; Zimmer et al. 2009; Laurent et al. 2015; Li et al. 2020). We hypothesized that FLP-14 engages multiple receptors in the O_2_ circuit to regulate O_2_-guided behaviors. To investigate this, we first examined if loss of *flp-14* alters aerotaxis in an O_2_ gradient. We used a gradient from 7% to 21% O_2_, because *C. elegans* dwell locally at 7% O_2_, whereas 21% O_2_ is aversive (Gray et al. 2004; Cheung et al. 2005; Rogers et al. 2006; Li et al. 2020). Animals roaming a thin bacterial lawn were exposed to the O_2_ gradient, and their behavioral responses recorded for 15 min. As a control strain, we used animals bearing a loss-of-function mutation in the neuropeptide receptor gene *npr-1* in all assays. We did not use the reference laboratory strain N2, because it has acquired a gain-of-function mutation in *npr-1* that diminishes aversion from 21% O_2_ in the presence of bacterial food (de Bono and Bargmann 1998; Gray et al. 2004; Cheung et al. 2005; McGrath et al. 2009; Persson et al. 2009). Initially, we tested animals reared at atmospheric (21%) O_2_ levels. While the median position of *flp-14* mutants was slightly lower than the median position of control animals on the gradient (13% reduction; Supplementary Fig. 1, a and b), we observed no significant difference in the normalized aerotaxis index (Supplementary Fig. 1c). Because avoidance of 21% O_2_ is enhanced in animals grown at low O_2_ levels (Cheung et al. 2005; Li et al. 2022), we also tested animals acclimated to 7% O_2_ for abnormal aerotaxis. As expected, *npr-1* animals avoided 21% O_2_, resulting in a negative aerotaxis index (Fig. 1, c to e). Mutants lacking *flp-14* displayed stronger avoidance than controls, reflected in both a stronger reduction in median position on the gradient (42% reduction; Fig. 1d) and a significant decrease in aerotaxis index (Fig. 1e). In addition, *flp-14* mutants seemed to move more quickly on the gradient, as most animals reached their preferred position after 0 to 5 min, while control animals only reached a stable aerotaxis index after 5 to 10 min (Fig. 1e).

When exposed to elevated O_2_ levels, *C. elegans* become hyperactive and enter an enduring state of locomotory arousal. During this state, they increase their persistence of forward travel by augmenting their speed of movement and suppressing spontaneous short reversals (Cheung et al. 2005; Rogers et al. 2006). The enhanced O_2_ avoidance of *flp-14* mutants prompted us to examine if loss of FLP-14 alters locomotory arousal in response to O_2_. We exposed *flp-14* mutants to stepwise increasing O_2_ concentrations and analyzed their speed of forward movement and frequency of reorientations by reversal and turning. Mutants of *flp-14* moved more rapidly in response to rising O_2_ levels (Fig. 1f), while their frequency of reversing and turning was not affected (Supplementary Fig. 1, e and f). These results suggest that FLP-14 opposes O_2_-evoked arousal when animals are acclimated to 7% O_2_. When acclimated to 21% O_2_, *flp-14* mutants moved slightly more slowly than controls at lower O_2_ concentrations, but the absolute speed at 21% O_2_ was not significantly different from *npr-1* animals (Supplementary Fig. 1, d, g, and h), which aligns with the indifferent aerotaxis index of strains cultivated at this condition (Supplementary Fig. 1c). These results suggest that FLP-14 signaling in aerotaxis and O_2_-evoked arousal is context-dependent, eliciting larger effects when animals are acclimated to a low O_2_ environment.

To validate that FLP-14 influences O_2_-evoked arousal, we tested 2 additional mutant strains, each harboring another *flp-14* deletion allele (Supplementary Fig. 1i). Both loss-of-function mutations enhanced rapid movement in response to O_2_ (Supplementary Fig. 1j). To show conclusively that loss of *flp-14* heightens O_2_ responses, we performed transgenic rescue experiments. Expressing a wild-type *flp-14* transgene restored the speed at aversive O_2_ levels in *flp-14* mutants (Fig. 1g). Conversely, animals overexpressing *flp-14* were less aroused by O_2_ than control animals (Fig. 1h), which corroborates an inhibitory role of *flp-14* in locomotory arousal.

Previous work identified a role for FLP-14 in habituation to noxious heat and harsh touch, in addition to enhancing acute responses to these stimuli (Marques et al. 2021; P.Z. Wang et al. 2025). We therefore asked if FLP-14 alters locomotory responses to repeated or prolonged O_2_ stimuli besides attenuating immediate responses. The effect size of the speed increase in *flp-14* mutants did not change with repeated stimulation (Supplementary Fig. 2a). We also increased the duration of exposure to 21% O_2_ from 2 to 30 min, but heightened arousal in *flp-14* mutants persisted throughout the interval (Supplementary Fig. 2b). We conclude that FLP-14 persistently opposes locomotory arousal by O_2_ and is not required for maintaining the arousal response.

### FLP-14 release from O_2_-sensing neurons and interneurons attenuates O_2_ responses

Through CRISPR-mediated insertion of GFP in the endogenous locus, we confirmed that the expression pattern of *flp-14* in *npr-1* animals (Fig. 2a) matches *flp-14*^*+*^ cells identified previously in the reference laboratory strain N2 (Sun and Hobert 2021). Among these, *flp-14* is expressed in the main O_2_-sensory neurons, URX and PQR, and the RMG interneurons, that signal 21% O_2_ (Fig. 2a) (Gray et al. 2004; Cheung et al. 2005; Rogers et al. 2006; Persson et al. 2009; Zimmer et al. 2009; Busch et al. 2012). Furthermore, *flp-14* is expressed in modulatory and command interneurons that control movement, such as RID, AVB, LUA, and PVC (Chalfie et al. 1985; Li et al. 2014; Kato et al. 2015; Lim et al. 2016; Nichols et al. 2017; Chew, Tanizawa, et al. 2018; Emmons 2024; Meng et al. 2024).

To gain insight into the cellular origin of FLP-14 in O_2_ responses, we focused on the increased locomotory arousal of *flp-14* mutants. Targeted expression of wild-type *flp-14* in the URX O_2_ sensors alone rescued the increased arousal of *flp-14* mutants, and expression in URX, AQR, and PQR partially restored the arousal phenotype (Fig. 2, b and c). O_2_-evoked arousal was also rescued by expressing *flp-14* under the *flp-5* promoter that drives expression in RMG and AQR, among other neurons (Fig. 2d and Supplementary Table 2). Restoring *flp-14* expression in RMG alone only partially rescued the increased locomotor activity of *flp-14* mutants (Fig. 2e). Besides neurons in the O_2_-sensing circuit, we also tested if *flp-14* can act in the RID and LUA interneurons, which regulate locomotion (Lim et al. 2016; Oren-suissa et al. 2016; Chew, Tanizawa, et al. 2018; Marques et al. 2026). Expressing *flp-14* in RID under the *ceh-10* promoter attenuated O_2_ responses in the *flp-14* mutant (Fig. 2f), while expression of *flp-14* in LUA did not restore speed at increased O_2_ levels (Fig. 2g). We also tested whether ectopic expression of *flp-14* in the head region is sufficient to restore O_2_ responses, by expressing *flp-14* in the pharyngeal muscles, but observed no rescue of the mutant hyperarousal (Fig. 2h). These results support that FLP-14 release from URX and PQR O_2_ sensors suppresses locomotory arousal by O_2_, and *flp-14* expression in 2 additional classes of neurons—RMG and RID—at least partially contributes to this effect.

### FLP-14 signals through 3 GPCRs—DMSR-3, FRPR-8, and NPR-40—with nonredundant roles in O_2_-evoked arousal

To gain insight into FLP-14's actions in the O_2_ circuit, we sought to identify target receptors that are important for FLP-14 signaling in attenuating O_2_-evoked arousal. Previous screens for receptor activation in heterologous expression systems identified 12 FLP-14 receptors with diverse phylogenetic relations among each other (NPR-1, NPR-4, NPR-6, NPR-11, NPR-39, NPR-40, DMSR-1, DMSR-3, DMSR-7, FRPR-8, FRPR-10, and FRPR-19) (Gershkovich et al. 2019; Beets et al. 2023; P.Z. Wang et al. 2025). To identify FLP-14 receptors involved in O_2_ responses, we examined loss-of-function alleles for 7 receptor genes: *npr-39*, *npr-40*, *dmsr-1*, *dmsr-3*, *dmsr-7*, *frpr-8*, and *frpr-19* (Fig. 3, Supplementary Fig. 3, a to c). These include all highest-potency receptors activated by FLP-14, with EC_50_ values in the submicromolar (<1 μM) range, and cover representatives for 3 RFamide receptor families (*npr-40* EF/YL/IRFamide family receptor, *dmsr* myosuppressin-like receptors, and *frpr* FMRFamide peptide family receptors) (Beets et al. 2023). Mutants for *npr-40, dmsr-3, frpr-8,* and *frpr-19* each recapitulated the *flp-14* mutant phenotype, moving faster than control animals in response to aversive O_2_ stimuli, whereas mutants for *npr-39*, *dmsr-1*, and *dmsr-7* showed normal arousal (Fig. 3, a to d, Supplementary Fig. 3, a to c). Expressing wild-type receptor gene copies restored O_2_-evoked arousal in *npr-40, dmsr-3,* and *frpr-8* mutants, while O_2_ responses of the *frpr-19* mutant were not rescued (Fig. 3, e to h). We conclude that at least 3 FLP-14 receptors from phylogenetically diverse RFamide receptor families—NPR-40, DMSR-3, and FRPR-8—are individually required to counteract hyperarousal by O_2_.

We next examined if *npr-40, dmsr-3,* and *frpr-8* act in the same genetic pathway as *flp-14*. Overexpressing *flp-14* reduced locomotory arousal in response to O_2_ (Fig. 1h); if this effect depends on *npr-40, dmsr-3,* or *frpr-8*, overexpression of *flp-14* should not affect O_2_ responses in mutants for these genes. Accordingly, *flp-14* overexpression had no effect on O_2_-evoked arousal of *dmsr-3* or *frpr-8* mutants (Fig. 4, a and b). Overexpressing *flp-14* in *npr-40* mutants reduced the absolute speed at 14% and 17% O_2_, but not at 21% O_2_ (Fig. 4c). This suggests either that *npr-40* acts independently from *flp-14* in O_2_ responses at 14% and 17% O_2_, or that *npr-40* acts downstream of *flp-14*, but *frpr-8* and *dmsr-3* are sufficient to decrease responses at these O_2_ concentrations. If *npr-40* acts downstream of *flp-14*, we expect double mutants of *flp-14* and *npr-40* to behave like single mutants. Indeed, we found no significant differences in the O_2_ responses of *flp-14;npr-40* animals and single mutants (Fig. 4d).

**Fig. 4.**
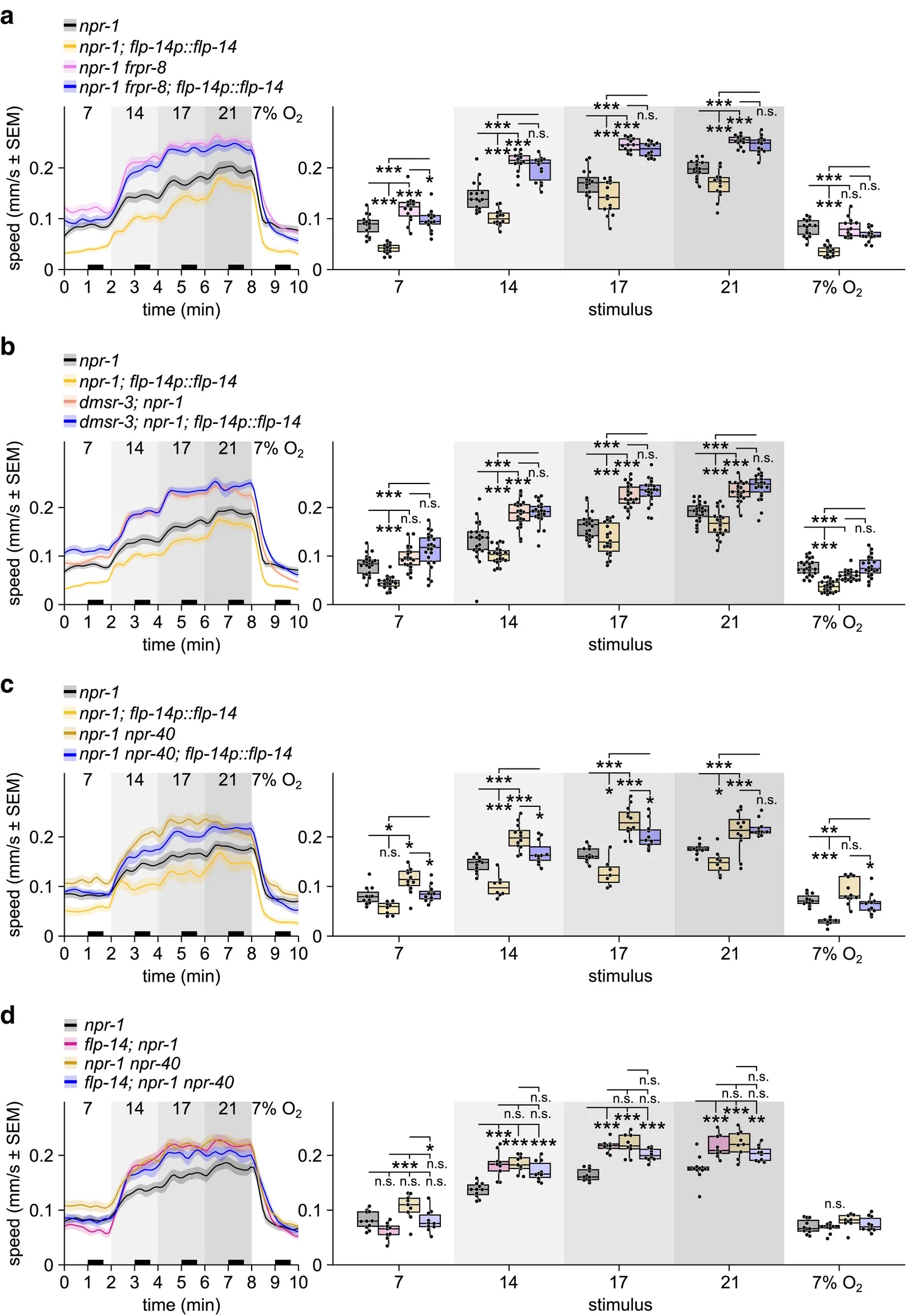
*frpr-8*, *dmsr-3*, and *npr-40* act downstream of *flp-14* in O_2_-evoked arousal. a and b) *flp-14* overexpression no longer reduces O_2_-evoked arousal in *frpr-8* and *dmsr-3* mutants. c) Overexpressing *flp-14* partially reduces speed at 14 and 17% O_2_ in *npr-40* mutants. d) Mutants lacking both *flp-14* and *npr-40* are not more aroused by O_2_ in comparison to *flp-14* and *npr-40* single mutants. Average speed over time is depicted as smoothed mean values (mm/s) ± SEM; *n* ≥ 8 on at least 3 d; intervals for which values were averaged in statistical comparison are indicated as black lines on the speed traces; ANOVA was performed on a GLMM considering paired measures per replicate and were corrected for all relevant, displayed comparisons using Holm–Bonferroni correction; *P*-values ns = not significant; * < 0.05; ** < 0.01; *** < 0.005.

Disrupting *dmsr-3*, *frpr-8*, or *npr-40* individually generated a hyperarousal phenotype comparable to the hyperactivity observed in the *flp-14* mutant (Fig. 3, a to c). We next asked whether these receptors act in the same genetic pathway by examining O_2_-evoked arousal in double and triple receptor mutants. Loss of either combination of 2 receptors as well as loss of all 3 receptors did not significantly alter hyperarousal compared to the single receptor mutants (Supplementary Fig. 4, a and b). Individual FLP-14 receptors thus attenuate locomotory arousal by acting independently rather than having overlapping functions. Taken together, these results indicate that at least 3 receptors act downstream of FLP-14 (DMSR-3, FRPR-8, and NPR-40), which have nonredundant roles and act in the same genetic pathway to attenuate O_2_-evoked arousal.

### Different FLP-14 receptors act in distinct neurons of the O_2_-sensing circuit to suppress locomotory arousal

In which neurons do FLP-14 receptors act to oppose arousal by O_2_? To probe this, we further examined the expression patterns of *dmsr-3*, *frpr-8*, and *npr-40* using fluorescent reporter strains. We constructed transcriptional reporter strains for each receptor gene by inserting a *gfp* transgene into the endogenous locus using CRISPR. To determine the identities of GFP-expressing neurons, we crossed each reporter strain with the NeuroPAL landmark strain that differentially labels individual neuron classes in the *C. elegans* nervous system (Yemini et al. 2021). Each receptor shows broad expression in multiple, partially overlapping neuron types across the nervous system. We identified 32 neuron types expressing *dmsr-3*, 16 neuron classes expressing *frpr-8*, and 13 neuron types that express *npr-40* (Fig. 5, a to c, Supplementary Fig. 5a). Because the expression patterns of *dmsr-3* and *frpr-8* overlap with neurons that express *flp-14*, we asked if these receptors autoregulate *flp-14* expression, by quantifying fluorescence of a transcriptional *flp-14* reporter in cells with overlapping receptor expression in *dmsr-3* and *frpr-8* mutants. We observed no difference in fluorescence compared to controls (Supplementary Fig. 5, b and c); thus, *dmsr-3* and *frpr-8* are not required to maintain *flp-14* expression cell-autonomously.

**Fig. 5.**
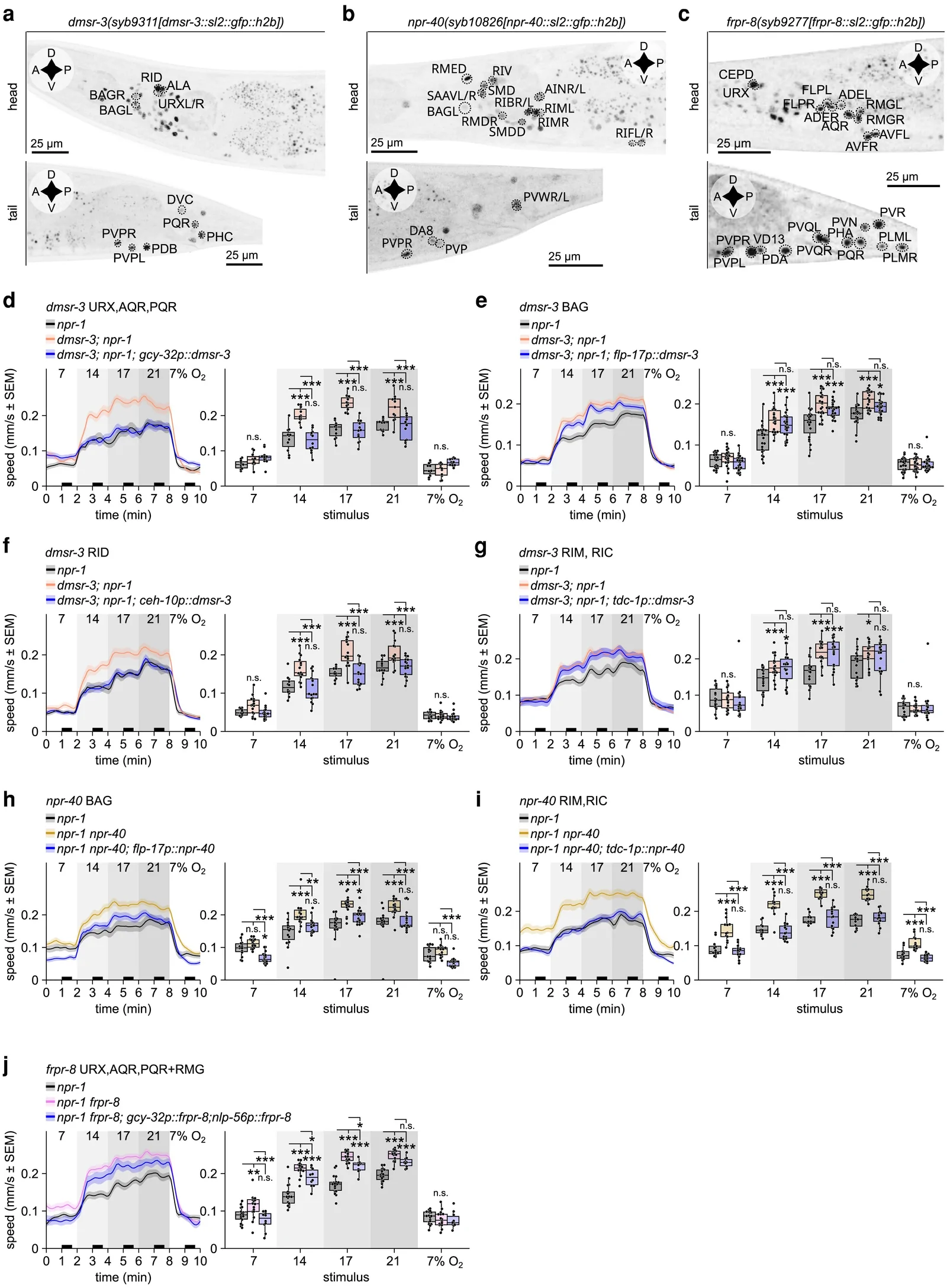
Distinct FLP-14 receptors act on different neurons in a distributed circuit for locomotory arousal. a to c) Expression patterns of transcriptional reporter strains for *dmsr-3* (also see Supplementary Fig. 5a), *npr-40,* and *frpr-8* in *npr-1* animals by CRISPR-insertion of a fluorescent reporter with nuclear localization in the endogenous locus (*sl2::gfp::h2b*). Animals were acclimated at 7% O*_2_*. D, dorsal; V, ventral; A, anterior; P, posterior. d to j) FLP-14 receptors act in different O_2_-sensory neurons (also see Supplementary Fig. 3, d and e): d and e) Restoring *dmsr-3* expression in URX, AQR, and PQR is sufficient to rescue locomotory arousal in *dmsr-3* mutants, whereas expression in BAG is not. f and g) Expression of *dmsr-3* in RID, but not in RIM/RIC, rescues O_2_-evoked arousal in *dmsr-3* mutants. h) Expression of *npr-40* in BAG is sufficient to rescue the *npr-40* mutant phenotype. i) Expression of *npr-40* in RIM/RIC restores locomotory arousal in *npr-40* mutants. j) Expression of *frpr-8* in URX, AQR, PQR, and RMG decreases hyperarousal in *frpr-8* mutants. See Supplementary Table 2 for an overview of promoter sequences and expression patterns. Average speed through time is depicted as smoothed mean values (mm/s) ± SEM; *n* ≥ 8 on at least 2 d; intervals for which values were averaged in statistical comparison are indicated as black lines on the speed traces. ANOVA was performed on a GLMM considering paired measures per replicate and were corrected for all relevant comparisons and testing of rescue strains targeting multiple neuronal populations across figure panels using Holm–Bonferroni correction; *P*-values ns = not significant; * < 0.05; ** < 0.01; *** < 0.005.

To identify neurons in which *dmsr-3*, *npr-40*, and *frpr-8* act to suppress O_2_-evoked arousal, we performed cell-specific rescue experiments. We first focused on neurons of the core circuit for O_2_-sensing, including the O_2_-sensory neurons URX, AQR, PQR, and BAG, and the RMG interneurons. O_2_ responses of *dmsr-3* mutants were rescued by restoring *dmsr-3* expression in URX and PQR, but not by restoring its expression in BAG (Fig. 5, d and e). By contrast, expressing *npr-40* in BAG neurons was sufficient to rescue arousal by O_2_ in *npr-40* mutants (Fig. 5h). DMSR-3 and NPR-40 thus act in distinct O_2_-sensing neurons to attenuate arousal by O_2_. Because *frpr-8* is expressed in URX, AQR, PQR, and RMG, we asked if *frpr-8* acts in these neurons to suppress O_2_ responses. Expressing *frpr-8* in all 4 neuron types partially rescued the enhanced O_2_ responses of *frpr-8* mutants (Fig. 5j). Expressing *frpr-8* in the O_2_-sensing neurons or in RMG alone did not rescue this phenotype (Supplementary Fig. 3, d and e), suggesting that *frpr-8* acts in multiple neuron classes.

Since our results show that FLP-14 signaling targets receptors in O_2_-sensing neurons as well as RMG to attenuate arousal by O_2_, we asked if *flp-14* influences O_2_-evoked Ca^2+^ activity in these neurons. However, ratiometric Ca^2+^ imaging at the soma of URX, RMG, or BAG was not altered by loss of *flp-14* (Supplementary Fig. 6, a to c). To explore if FLP-14 signaling acts downstream of Ca^2+^ signaling, we quantified expression of the neuropeptide genes *flp-8* and *flp-17,* whose transcription is regulated by URX and BAG activity, respectively (Cheung et al. 2005; Laurent et al. 2015; Hussey et al. 2018; Li et al. 2020). While we observed no difference in expression of a *flp-17* reporter in BAG (Supplementary Fig. 6d), *flp-8* expression in URX was reduced in *flp-14* mutants (Supplementary Fig. 6, e and f), which could be partially rescued by expressing wild-type *flp-14* (Supplementary Fig. 6g). We also examined whether *flp-8* expression is influenced by FLP-14 receptors expressed in URX and observed a significant decrease in URX *flp-8* expression in mutants of *dmsr-3* (Supplementary Fig. 6h). Together, these findings suggest that FLP-14 signaling stimulates *flp-8* expression in URX.

To investigate if a reduction of *flp-8* transcription underlies hyperarousal of *flp-14* mutants, we increased *flp-8* transcripts again by transgenic expression in *flp-14* mutants. However, *flp-8* overexpression was not sufficient to attenuate hyperarousal in *flp-14* mutants (Supplementary Fig. 6i). Other manipulations revealed more complex genetic interactions between the 2 neuropeptide genes. Like *flp-14* mutants, *flp-8* mutants displayed hyperarousal, yet *flp-14;flp-8* double mutants were less aroused by O_2_ than the single mutants (Supplementary Fig. 6j). We conclude that *flp-8* and *flp-14* are both involved in attenuating arousal through partially antagonistic mechanisms. Together, these results indicate that loss of *flp-14* alters *flp-8* neuropeptide expression in URX, but this is not sufficient to explain hyperarousal by O_2_ in *flp-14* mutants.

### FLP-14 signaling additionally targets modulatory interneurons through different receptors

Our results showed that FLP-14 signaling targets distinct O_2_-sensing neurons, URX and BAG, through different receptors. Besides sensory neurons, we also observed strong expression of *dmsr-3* and *npr-40* in interneurons, called RID and RIM (Fig. 5, a and b, Supplementary Fig. 5a), which coordinate locomotor responses to aversive stimuli and have antagonistic roles in forward movement (Meisel et al. 2014; Chew, Tanizawa, et al. 2018; Marquina-Solis et al. 2024). RID is a neuroendocrine neuron that promotes the forward motor state, whereas tyraminergic RIM interneurons play a central role in reversal regulation (Gordus et al. 2015; Kato et al. 2015; Lim et al. 2016; Nguyen et al. 2016; Venkatachalam et al. 2016; Sordillo and Bargmann 2021). We hypothesized that FLP-14 signaling in O_2_-evoked arousal is distributed across multiple circuit layers, including sensory neurons and interneurons, and requires coordinated modulation of RID and RIM through different receptors. Indeed, restoring *dmsr-3* expression in RID using the *ceh-10* promoter rescued O_2_-evoked arousal of *dmsr-3* mutants (Fig. 5f), while cell-specific expression of *npr-40* in tyraminergic neurons rescued arousal in *npr-40* mutants (Fig. 5i). By contrast, expression of *dmsr-3* in tyraminergic neurons did not rescue hyperarousal of the *dmsr-3* mutant (Fig. 5g). Together, these data suggest that FLP-14 peptides act on different receptors in distinct neurons that mediate O_2_ sensing and motor control to counteract O_2_-evoked arousal.

## Discussion

Chemical messengers, including neuropeptides, diversify their actions by binding to multiple receptors. Receptor diversity is often ascribed to functional redundancy or pleiotropic functions of neuropeptides (Boonen et al. 2009; Nässel and Wegener 2011; Ikeda et al. 2013; Pedragosa-badia et al. 2013; Elphick et al. 2018; Pu et al. 2023; Wang et al. 2023; Hevesi et al. 2024; Marquina-Solis et al. 2024). How promiscuous peptides act on different receptors within neural circuits is, however, not well understood (Williams et al. 2017; Smith et al. 2019; Melzer et al. 2021; Hevesi et al. 2024; Jékely and Yuste 2024; Beets and Watteyne 2025). Here, we show that the *C. elegans* RFamide neuropeptide FLP-14 attenuates arousal in response to O_2_ by signaling to a distributed network of phylogenetically diverse receptors, which have congruent but nonredundant roles in locomotory arousal.

Together with previous work (Meisel et al. 2014; Gordus et al. 2015; Kato et al. 2015; Lim et al. 2016; Nguyen et al. 2016; Venkatachalam et al. 2016; Sordillo and Bargmann 2021; Marquina-Solis et al. 2024), our results suggest a model in which FLP-14 signals through different receptors to modulate distinct and functionally opposing neurons of the locomotory arousal circuit (Fig. 6). Aversive O_2_ levels evoke FLP-14 release primarily from the O_2_ sensory neurons URX and PQR, as well as from downstream recruited interneurons RMG and RID. FLP-14 acts on DMSR-3, NPR-40, and FRPR-8 across sensory neurons and interneurons, including URX, BAG, RMG, RID, and RIM, consistent with the distributed topology of neuromodulatory networks (Smith et al. 2019; Marques et al. 2021; Ripoll-Sánchez et al. 2023; Salvan et al. 2023; Marquina-Solis et al. 2024; Feng et al. 2025). Disrupting each receptor evokes hyperarousal comparable to disrupting *flp-14*. Taken together, our data suggest that FLP-14 signaling requires coordinated modulation of multiple, distributed circuit nodes through distinct receptors. These include neurons that display increases (RMG and RID) and decreases (RIM) in activity during forward locomotion (Gordus et al. 2015; Kato et al. 2015; Lim et al. 2016; Nguyen et al. 2016; Venkatachalam et al. 2016; Sordillo and Bargmann 2021).

**Fig. 6.**
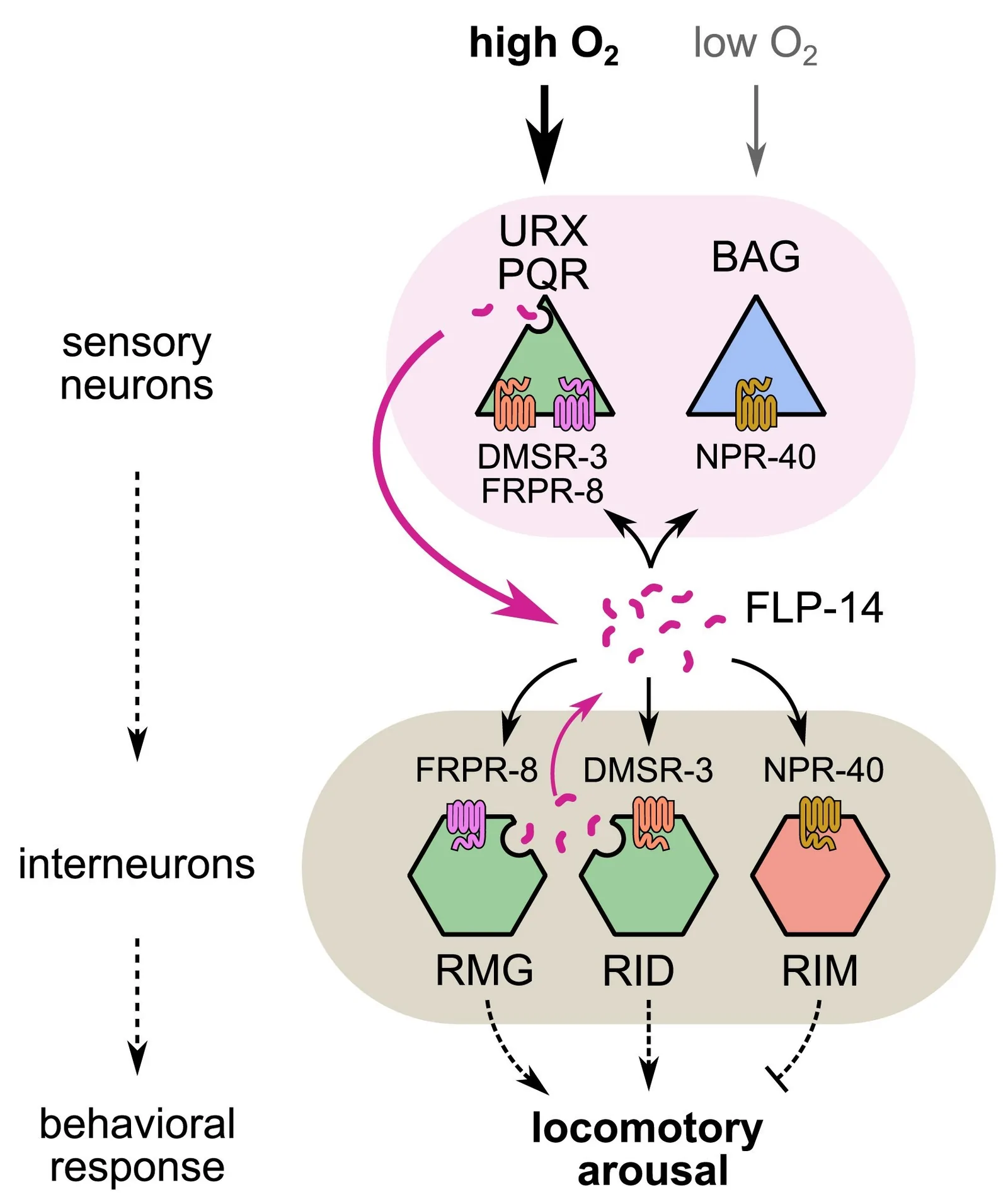
A model for FLP-14 signaling in O_2_-evoked locomotory arousal. Upon exposure to aversive O_2_, sensory neurons URX and PQR, and connected RMG interneurons, are activated and recruit downstream locomotor interneurons, including RID, that collectively initiate locomotory arousal. Activation of URX and PQR, together with RMG and RID, results in the release of FLP-14 from these neurons, which in turn modulates distributed neurons through different receptors to attenuate locomotory arousal. FLP-14 signaling acts on DMSR-3 in URX and RID, on FRPR-8 in O_2_ sensory neurons and RMG, and on NPR-40 in BAG and RIM. In our data, DMSR-3 and NPR-40 act distinctively on separate target neurons, including forward-promoting (→) RID neurons and forward-suppressing (⊣) RIM neurons.

Our findings expand the receptor complement that is recruited by FLP-14 in vivo, as we show that *dmsr-3*, *npr-40*, and *frpr-8* act downstream of *flp-14* in attenuating O_2_-evoked arousal. Overall, the functional organization of the FLP-14 RFamide system resembles the organization of aminergic neuromodulatory networks, which display extensive ligand promiscuity and context-dependent effects on behavior by engaging multiple, phylogenetically diverse receptors (Carre-Pierrat et al. 2006; Yamamoto et al. 2012; Tierney 2018; Florman and Alkema 2022; Salvan et al. 2023; Feng et al. 2025). We find that FLP-14 attenuates O_2_-evoked arousal through at least 3 GPCRs (DMSR-3, FRPR-8, and NPR-40) that belong to diverse protostomian and nematode families of RFamide receptors (Jékely 2013; Mirabeau and Joly 2013; Beets et al. 2023). These receptors differ from those relevant for FLP-14 in salt chemotaxis learning, which requires *frpr-10* but not *dmsr-3*, *frpr-8*, or *npr-40,* nor *frpr-19* that regulates nociceptive responses (Marques et al. 2021; P.Z. Wang et al. 2025). Furthermore, our results highlight context-dependent effects, showing opposite actions of *flp-14* in forward movement depending on the environmental and genetic context of the animals. A previous study showed that FLP-14 is released from RID to promote forward movement during foraging (Lim et al. 2016). This effect was observed in the laboratory reference strain N2, which has acquired a mutation in *npr-1* that confers O_2_-response defects (de Bono and Bargmann 1998; Coates and de Bono 2002; Macosko et al. 2009; Rockman and Kruglyak 2009; Weber et al. 2010; Carrillo et al. 2013; Kodama-Namba et al. 2013 ). By contrast, we find that FLP-14 release from O_2_-sensing neurons, RMG, and RID suppresses forward movement in *npr-1* animals when acclimated to low (7%) O_2_ concentrations. When acclimated to high (21%) O_2_, FLP-14 has only a minor, promoting effect on O_2_-evoked arousal. Differences in the sensory environment, genetic backgrounds, and underlying neural circuits may explain the distinct, opposite effects of FLP-14 signaling in these conditions (Bargmann 2012; Marder 2012; Fenk and de Bono 2017; Li et al. 2020; Beets and Watteyne 2025).

Through expression mapping, followed by rescue in candidate target neurons, we show that *dmsr-3*, *npr-40,* and *frpr-8* are broadly expressed and can act in at least 5 classes of neurons—URX, BAG, RMG, RID, and RIM—to attenuate arousal by O_2_. We find that different FLP-14 receptors modulate distinct, including functionally opposing, neurons. NPR-40 acts in BAG and RIM, whereas DMSR-3 is required in URX and RID to attenuate O_2_-evoked arousal. *dmsr-3* is also expressed in BAG and RIM, but hyperarousal could not be rescued by restoring expression in these neurons; thus, NPR-40 and DMSR-3 do not act interchangeably. Because RID and RIM have antagonistic actions in locomotion, our results suggest that FLP-14 opposes arousal by simultaneously regulating forward-promoting neurons (RID) through DMSR-3 and modulating forward-suppressing neurons (RIM) through NPR-40 (Meisel et al. 2014; Gordus et al. 2015; Kato et al. 2015; Lim et al. 2016; Nguyen et al. 2016; Venkatachalam et al. 2016; Chew, Grundy, et al. 2018; Sordillo and Bargmann 2021; Marquina-Solis et al. 2024).

The molecular mechanisms by which FLP-14 receptors affect their target neurons remain to be understood. Because loss of *dmsr-3* and *npr-40* both confers hyperarousal, we speculate that DMSR-3 acting in forward-promoting neurons is likely inhibitory, while NPR-40 acting in forward-inhibiting neurons presumably has an excitatory role. Our data suggest that *flp-14* modulates neuronal activity downstream or in parallel of Ca^2+^ responses, possibly by altering neuropeptide transmission. Loss of *flp-14* or *dmsr-3* reduced *flp-8* expression in URX, but this is not sufficient to explain the hyperarousal of *flp-14* mutants, because restoring *flp-8* expression did not rescue arousal. Instead, *flp-14* and *flp-8* display more complex genetic interactions. Disrupting each individually leads to hyperarousal, but simultaneous loss of *flp-14* and *flp-8* nearly restores normal arousal. We speculate that this interaction results from receptor cross-activity between FLP-14 and FLP-8 peptides, which bind multiple peptide-specific as well as overlapping receptors (Beets et al. 2023).

Across metazoans, ligand promiscuity has commonly been described for RFamide-like peptides (Walker et al. 2009; Li and Kim 2014; Peymen et al. 2014; Zatylny-Gaudin and Favrel 2014; Ma et al. 2017; Ubuka and Tsutsui 2018; Kovács et al. 2024). Such ligand promiscuity often underlies functional redundancy or pleiotropic actions in behavior (Cohen et al. 2009; Pedragosa-badia et al. 2013; Stawicki et al. 2013; Bhardwaj et al. 2018; Oranth et al. 2018; Marques et al. 2021; Pu et al. 2023; Hevesi et al. 2024; Marquina-Solis et al. 2024; Feng et al. 2025). In addition, we find that promiscuous FLP-14 signaling enables coordinated modulation of distributed circuit elements through different receptors. We expect such a topology-function relationship to extrapolate to other RFamide systems. In mammals, for example, PrRP binds to 6 receptors, including ligand-specific and promiscuous receptors of diverse receptor families (Jékely 2013; Mirabeau and Joly 2013; Foster et al. 2019; Gershkovich et al. 2019; Beets et al. 2023). Like FLP-14, different PrRP receptors show overlapping expression in several brain areas, and multiple PrRP receptors are thought to coordinately regulate stress and metabolism-related behaviors (Vas et al. 2023; Kovács et al. 2024; Y. Wang et al. 2025; Wu et al. 2025). Besides RFamide peptides, ligand promiscuity has been identified in other neuromodulatory systems as well, such as aminergic systems (Carre-Pierrat et al. 2006; Yamamoto et al. 2012; Pándy-Szekeres et al. 2018; Tierney 2018; Foster et al. 2019; Salvan et al. 2023; He et al. 2024). We speculate that ligand promiscuity evolved as a convergent signaling motif that may have been selected for pleiotropy, functional redundancy, as well as increased bandwidth to differentially modulate distributed circuit elements.

Neuropeptide GPCRs have become attractive therapeutic targets (Komatsu 2015; Hauser et al. 2017; Foster et al. 2019). At the same time, both drawbacks and advantages of targeting multireceptor signaling systems have been acknowledged (Ehrt et al. 2019). Our work underscores the multisite actions of promiscuous peptides in behavior. This supports the necessity for system-wide studies toward the biological actions of endogenous multireceptor ligands.

## Supplementary Material

iyag188_Supplementary_Data

## Data Availability

The data underlying this article are available in the article and in its online supplemental material. Strains and plasmids are available upon request. Supplemental material available at GENETICS online.
