# Supplementary material for: Distinct receptors for a promiscuous RFamide neuropeptide co-regulate a distributed circuit for locomotory arousal in C. elegans
Source: Genetics. 2026 Jul 18;234(3):iyag188. doi: 10.1093/genetics/iyag188 (PMC13732711; doi:10.1093/genetics/iyag188)
Supplement: Supplementary file 1 [file iyag188_supplementary_data.zip › Supplementary_Figures_1-6_GENETICS-2026-309701.pdf]

Supplementary Material for

De Fruyt N, Chudinova A, Thapliyal S, Geens E, Roy C, Vandewyer E, Van Damme S, Mergan L, Beets I. 2026. Distinct receptors for a promiscuous RFamide neuropeptide co-regulate a distributed circuit for locomotory arousal in *C. elegans*. GENETICS.

Supplementary Figures and Figure Legends 1-6
Supplementary Table Legends 1-2

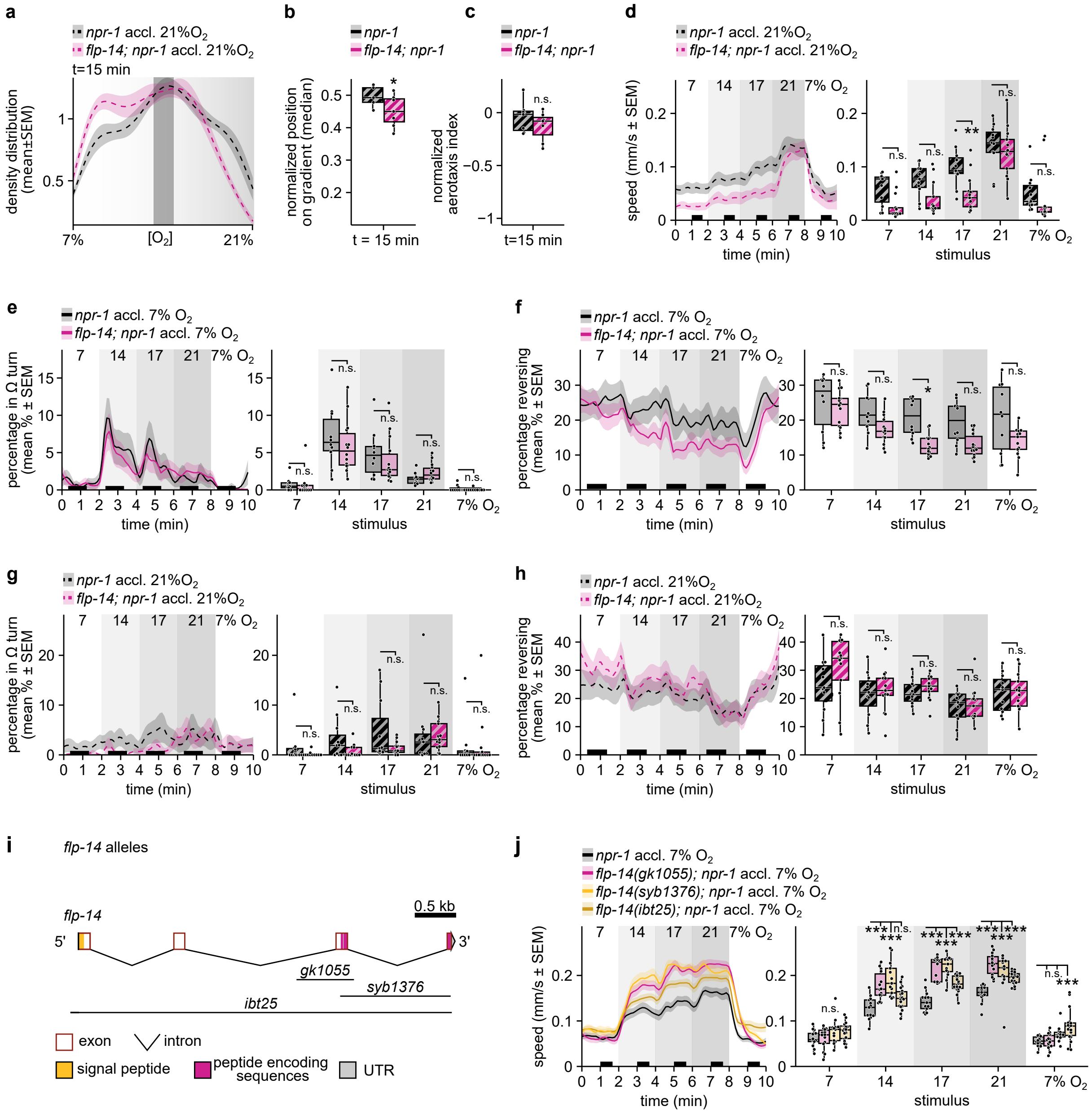

Supplementary Figure 1 – Behavioral responses of *flp-14* mutants in response to aversive O_2. a-c) Loss of *flp-14* does not affect aerotaxis when animals are acclimated to 21% O_2. **b)** The median position of *flp-14* mutants on a 7% to 21% O_2 gradient is slightly lower compared to controls when animals are acclimated to 21% O_2. The distribution of animals across the gradient is represented as average density across replicates ± SEM. **c)** The normalized aerotaxis index is not significantly different between *flp-14* mutants and *npr-1* controls. Aerotaxis index = ((number of animals at high O_2) – (number of animals at low O_2)) / (total number of animals), excluding animals at the origin (shaded region); indexes are normalized to the aerotaxis index at t = 0 min. **d)** The absolute speed of *flp-14* mutants at aversive O_2 concentrations is not significantly different from that of controls when acclimated to 21% O_2. Effect sizes and significances are variable throughout assays (data in additional assays not shown). **e-f)** *flp-14* mutants acclimated to 7% O_2 show nearly no differences in comparison to control animals in the proportion of animals reversing or

performing omega turns. **g-h)** *flp-14* mutants acclimated to 21% O_2 show normal reversal and turning behavior in response to O_2. **i)** Exon-intron scheme of *flp-14* loss-of-function alleles. *flp-14(gk1055)* was used in all figures unless stated otherwise (Supplementary Fig. 1j). **j)** O_2-evoked arousal of different *flp-14* mutants. Locomotion speed and the percentage of animals reversing or turning through time are depicted as smoothed mean values ± SEM; n ≥ 6 assays on at least 2 days; intervals for which values were averaged in statistical comparison are indicated as black lines on the trace plots; ANOVA was performed on a GLMM considering paired measures per replicate and were corrected for all relevant comparisons using Holm-Bonferroni correction; p-values n.s. = not significant; * < 0.05; ** < 0.01; *** < 0.005.

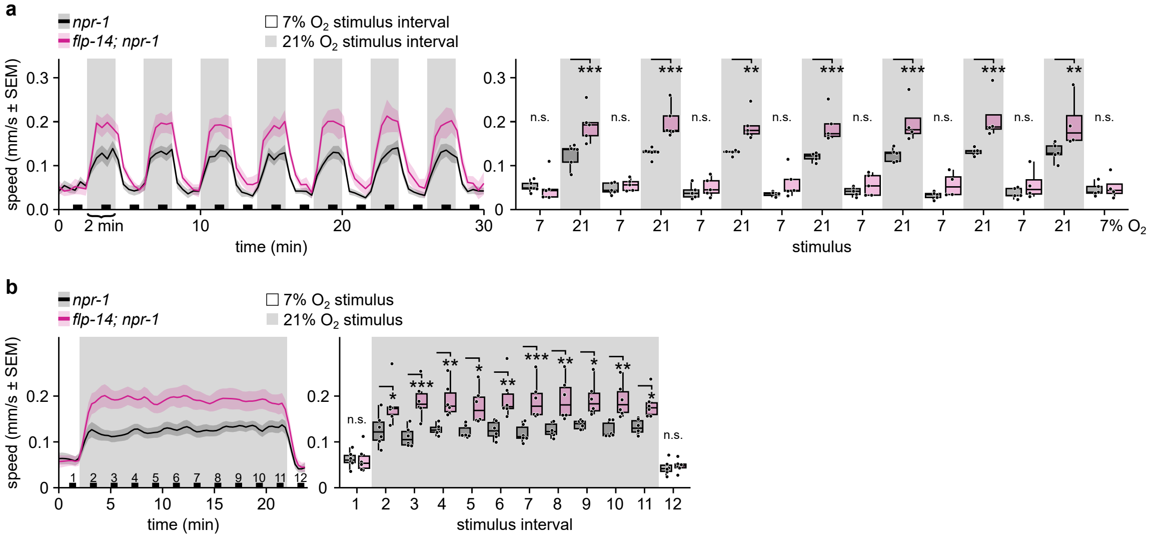

Supplementary Figure 2 – FLP-14 does not affect behavioral responses to repeated or prolonged O_2 stimuli. a) Repeated or **b)** prolonged stimulation with 21% O_2 does not affect O_2 responses of *flp-14* mutants in comparison to *npr-1* controls. Locomotion speed through time is depicted as smoothed mean values ± SEM; n ≥ 6 assays on at least 2 days; intervals for which values were averaged in statistical comparison are indicated as black lines on the trace plots; ANOVA was performed on a GLMM considering paired measures per replicate and were corrected for all relevant comparisons using Holm-Bonferroni correction; p-values n.s. = not significant; * <0.05; ** < 0.01; *** < 0.005.

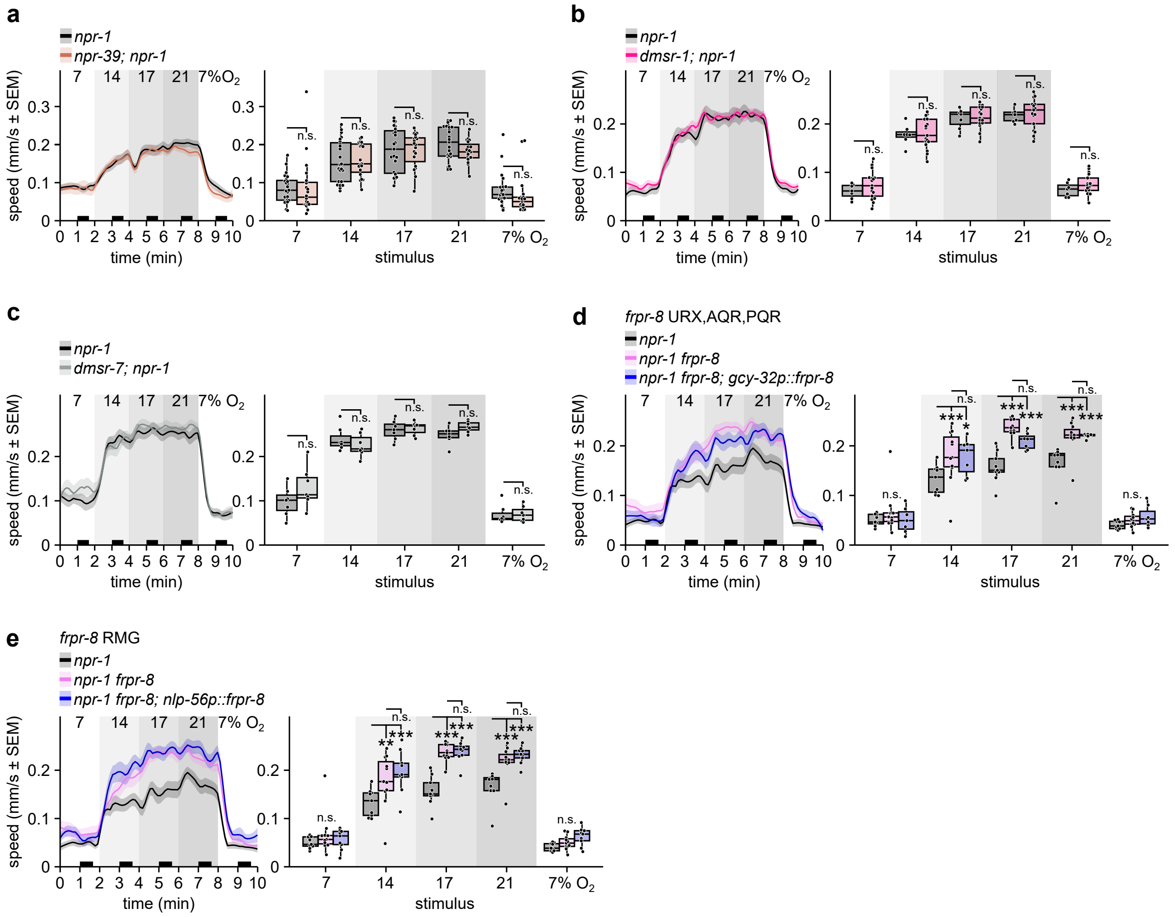

Supplementary Figure 3 – Mutants for *npr-39, dmsr-1* and *dmsr-7* do not recapitulate the *flp-14* mutant phenotype in O_2-evoked arousal. a-c) O_2-evoked arousal of *npr-39, dmsr-1,* and *dmsr-7* mutants is not significantly different from *npr-1* control animals. **d-e)** Related to Fig. 5. Expression of *frpr-8* in O_2-sensing neurons (URX, PQR and AQR), or in RMG alone does not restore locomotory arousal in *frpr-8* mutants. Animals were acclimated to 7% O_2 before the assay. Average speed over time is depicted as smoothed mean values (mm/s) ± SEM; n ≥ 7 on at least 2 days; intervals for which values were averaged in statistical comparison are indicated as black lines on the speed traces; ANOVA was performed on a GLMM considering paired measures per replicate and were corrected for all relevant comparisons and testing of multiple receptors, or multiple cellular sites across figure panels using Holm-Bonferroni correction; p-values n.s. = not significant; * <0.05; ** < 0.01; *** < 0.005.

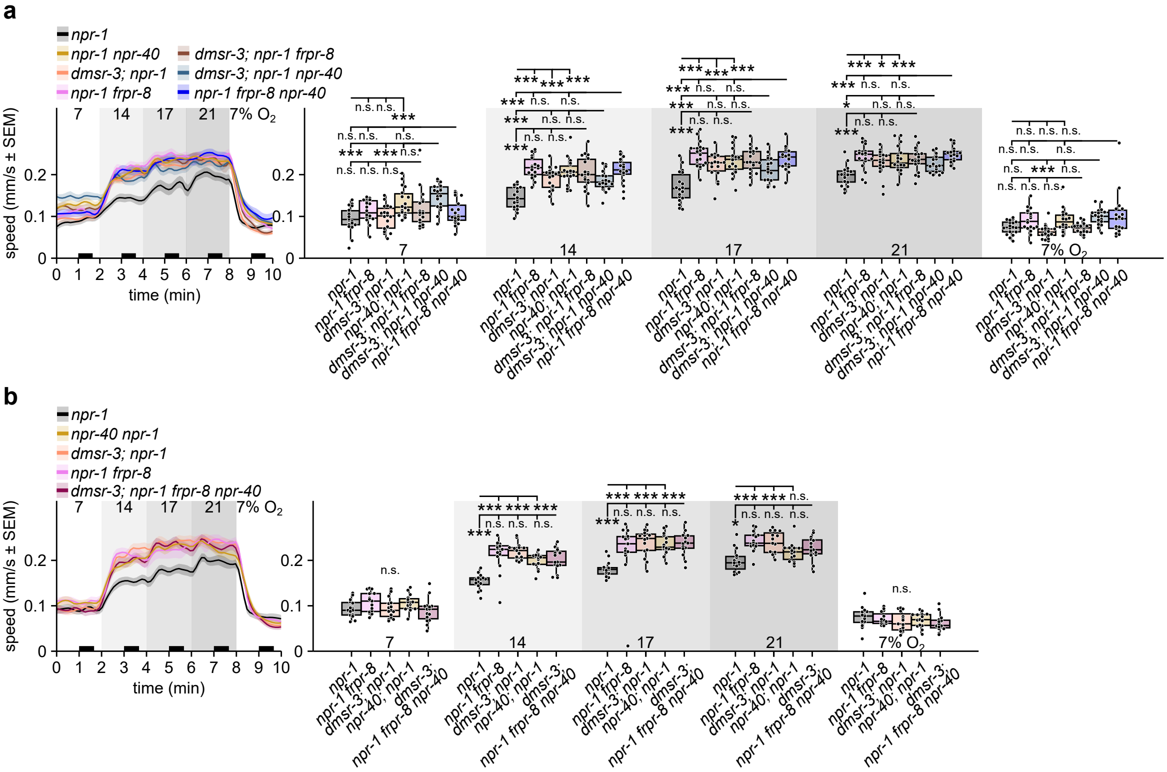

Supplementary Figure 4 – Simultaneous loss of *dmsr-3, npr-40* and *frpr-8* does not aggravate hyperarousal. a) Receptor double or **b)** triple mutants do not show additive effects compared to receptor single mutants. Average speed over time is depicted as smoothed mean values (mm/s) ± SEM; n ≥ 15 assays on at least 5 days; intervals for which values were averaged in statistical comparison are indicated as black lines on the speed traces; ANOVA was performed per receptor combination on a GLMM considering paired measures per replicate and were corrected for all indicated comparisons of each combinatorial mutant using Holm-Bonferroni correction; p-values n.s. = not significant; * <0.05; ** < 0.01; *** < 0.005.

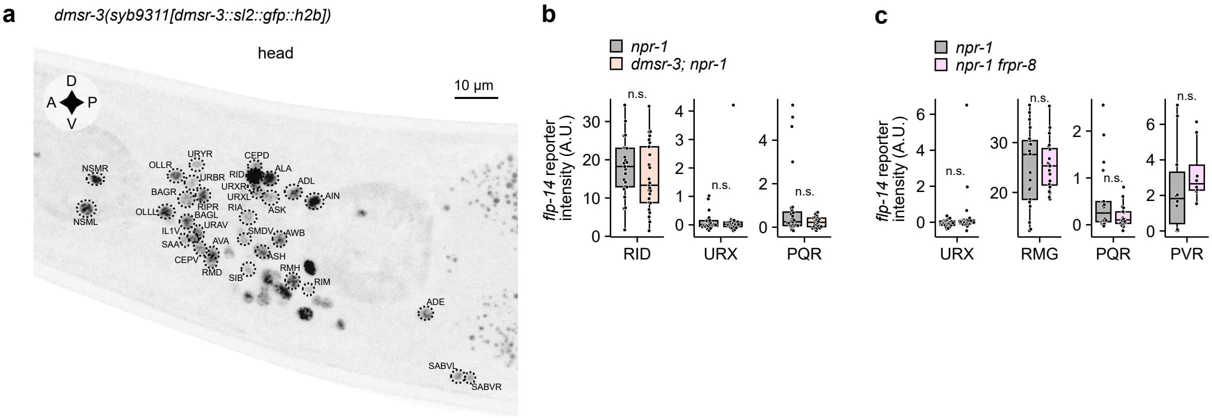

Supplementary Figure 5 – Loss of *dmsr-3* and *frpr-8* does not influence *flp-14* expression. **a)** *dmsr-3* expressing neurons in the head ganglia, complementary to Fig. 5a. D, dorsal; V, ventral; A, anterior; P, posterior. **b-c)** There are no differences in *flp-14* transcriptional reporter intensity in neurons that co-express *flp-14* and *frpr-8* or *dmsr-3* in respective receptor mutants. n ≥ 23 animals on 6 ≤ days; Significance determined by Kruskal-Wallis Rank Sum test with Wilcoxon-Wilk pairwise post-hoc testing, adjusted for multiple comparisons by Holm-Bonferroni correction; A.U. = arbitrary units; p-values n.s. = not significant.

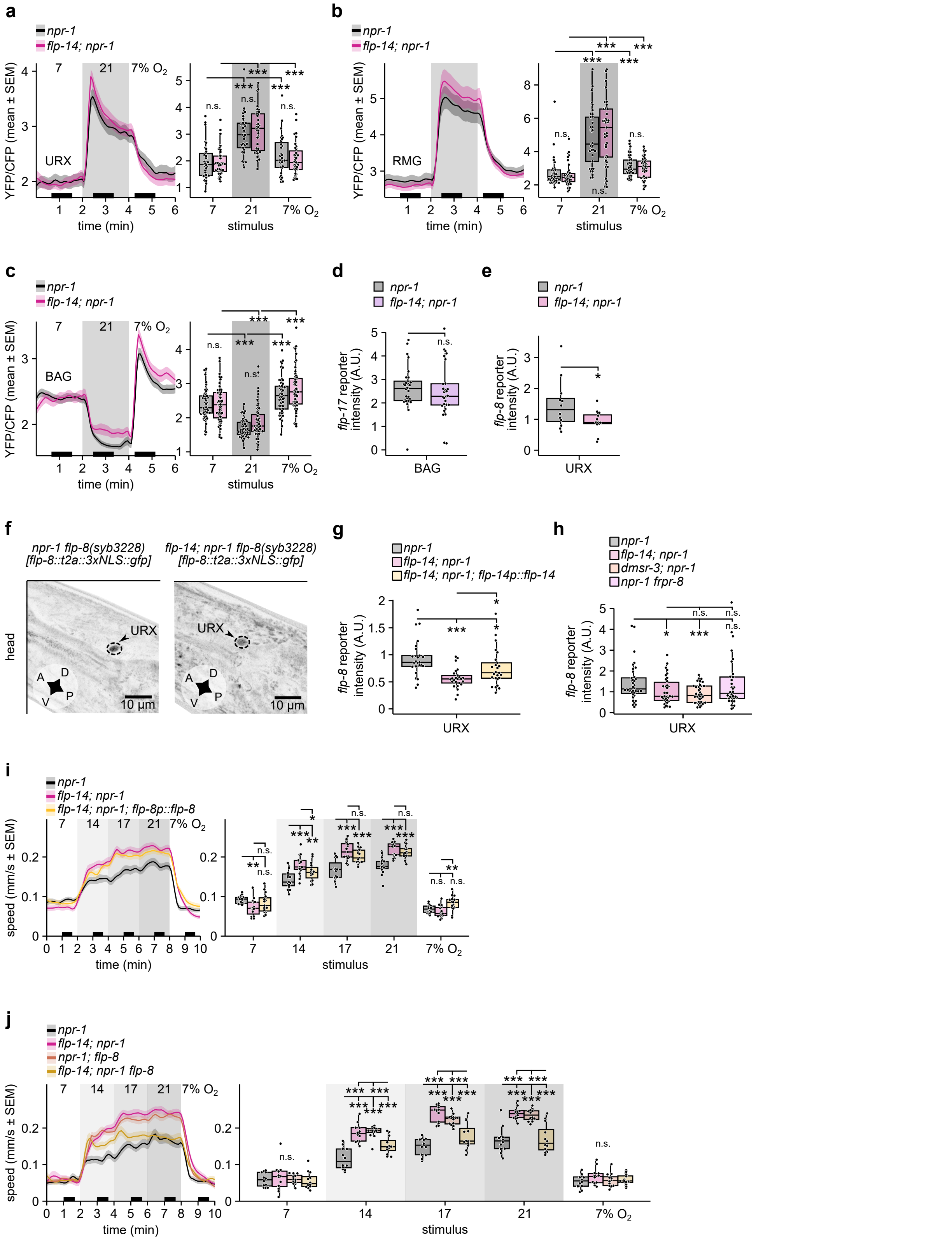

Supplementary Figure 6 – FLP-14 receptors act downstream of Ca^{2+} in the O_2-sensing neurons. a-b) O_2-evoked Ca^{2+} activity of URX and RMG neurons is not altered by loss of *flp-14*; n ≥ 39 animals collected on 10 assay days. **c)** BAG Ca^{2+} responses of *flp-14* mutants differ from those in *npr-1* controls (p-value = 0.02), but pairwise significance is lost after correcting for multiple comparisons (p-value = 0.06); n ≥ 51 animals on 8 assay days.

Intervals for which YFP/CFP ratios were averaged in statistical comparison are indicated as black lines in the speed over time panels; to meet normality criteria, YFP/CFP ratios were log(1+x) transformed. **d)** Expression of an endogenous, transcriptional *flp-17* reporter (*sl2::gfp::h2b*) does not differ in BAG neurons of *flp-14* mutants as compared to controls; n = 28 animals on 2 days. **e)** *flp-14* mutants show reduced expression of a translational reporter in the endogenous *flp-8* locus (*t2a::3xNLS::gfp*) in URX neurons; n = 12 animals, imaged on 2 days. **f)** Representative images illustrating a reduction in *flp-8* expression in URX neurons of *flp-14* mutants compared to *npr-1* animals. **g)** *flp-14* transgene expression can partially rescue *flp-8* expression levels in *flp-14* mutants; n = 25 animals on 3 days. **h)** *dmsr-3* mutants replicate decreased *flp-8* expression. Results are variable and inconclusive for *frpr-8* mutant animals; n ≥ 32 animals on 4 days. **i)** Enhancing *flp-8* expression is not sufficient to rescue locomotory arousal in *flp-14* mutants; n = 15 assays on 5 days. **j)** *flp-8* mutants phenocopy the enhanced O2-evoked arousal of *flp-14* mutants, whereas loss of *flp-8* in the *flp-14* mutant nearly abolishes the *flp-14* mutant phenotype; n = 12 assays on at least 3 days; *flp-17* and *flp-8* expression levels were compared by student t-test; to meet normality criteria, rescue of *flp-8* expression and *flp-8* expression in receptor mutants was compared by ANOVA on log(1+x) transformed values; locomotion speed through time is depicted as smoothed mean values ± SEM; ANOVA was performed on a GLMM considering paired measures per replicate and were corrected for all relevant comparisons using Holm-Bonferroni correction; p-values n.s. = not significant; * <0.05; ** < 0.01; *** < 0.005.

Supplementary Table Legends

Supplementary Table 1: Reagent Table
Supplementary Table 2: Promoter lengths and targeted cell types.
